# Analysis of hydraulic mechanism of dynamics flow visualization in an axial pump with impeller blades based on novel transient characteristics conditions and vibration techniques

**DOI:** 10.1038/s41598-026-36822-6

**Published:** 2026-01-27

**Authors:** Ahmed Ramadhan Al-Obaidi, Anas Alwatban

**Affiliations:** 1 Department of Mechanical Engineering, College of Engineering, Mustansiriyah, University, Baghdad, Iraq; 2 Department of Mechanical Engineering, College of Engineering, Qassim University, Qassim, 51452 Kingdom of Saudi Arabia

**Keywords:** Transient flow, Pressure fluctuations, Angle of blade, Simulation, Axial pump, Modeling, Energy science and technology, Engineering, Physics

## Abstract

Pumps operated in turbine mode have attracted considerable attention for hydropower generation and water conveyance applications due to their economic advantage over conventional hydroturbines. Despite this benefit, their deployment remains constrained by limited flow controllability and pronounced instability when operating away from the design point. To address these challenges, the present work combines experimental measurements with numerical simulations to examine the unsteady flow behavior of an axial-flow pump under five distinct operating regimes, spanning deep part-load conditions at 5 L/min through the design point and into overload operation at 12.5 L/min. Pump stability was evaluated through detailed analyses of velocity distributions and pressure fluctuations in both the time and frequency domains. The results reveal a strong dependence of unsteady behavior on operating condition. At part-load operation, pressure pulsations intensify markedly, with peak-to-peak amplitudes increasing by as much as 15% relative to the design flow rate. Spectral analysis shows that rotor–stator interaction phenomena dominate the unsteady response, with the blade passing frequency and its harmonics contributing over 12% of the total spectral energy across most monitoring locations. As the flow rate approaches overload, the magnitude of pressure oscillations is reduced by approximately 14%, indicating a progressive improvement in hydraulic stability. The effect of impeller blade stagger was further investigated for three configurations, namely − 3°, 0°, and + 3°. Deviations from the baseline geometry (0°) significantly amplify flow unsteadiness, particularly in the rotor–stator interaction region. In these cases, pressure pulsation amplitudes increase by up to 16%, highlighting the sensitivity of unsteady flow structures to blade-angle modification. Overall, the findings demonstrate that both operating regime and impeller blade angle exert a decisive influence on the stability and dynamic performance of axial-flow pumps, offering valuable insights for their optimal design and operation under variable flow conditions.

## Introduction

The well recognized technology of hydraulic pumps was used in energy generating systems, offering several benefits that primarily increase the viability of energy supply projects in isolated areas that was electric grid’s reach^1^. Furthermore, this technique may was utilized to reduce water distribution networks’ pressure levels while simultaneously generating useable electricity, thus saving a significant amount of energy^2^. Pumps perform better in one key area, as stated in several published works: cost-effectiveness^3^. Otherwise, its adoption would be hampered by technical flaws related to pumps, such as the intolerance of operating circumstances that are not part of the design or the well-known inability to accurately convert between pumping performance parameters^4^. Accordingly, a number of ongoing investigations were conducted to address the technological and scientific problems that are now present in both pump working modes^5^. Research on pump flow instability at changing conditions as well the corresponding formation mechanism of flow structure was conducted in this regard, in addition to studies that predict pump performance^6^. Over time, this should improve the performance pump’s balanced for mode of operating. A decrease in pump mechanical efficiency was observed under part-load situations due to flow separation and whirling flows inside the machine flow channels, according to newly published information on the same topic^7^.

Accordingly, several researches were conducted on an ongoing basis to solve the technoscientific problems that are now present in both of the pump’s working modes^8^. Research on pump flow instability at varying conditions as well the corresponding structure of flow formation was conducted for this regard, in addition to studies that predict pump performance^9^. Over time, this should improve the pump performance. A decrease in pump’s mechanical efficiency under part-load situations was shown in the newly published details on the same issue. This is due to flow separation and whirling flows inside the machine flow channels^10^. According to their research, the turbine mode of the pump outperforms pump through 30 and 39%, for BEP flow rate (QBEP) and Head. In the same vein, Shi et al.^11^ identified diffuser and impeller as primary domain flow with highest losses of mechanical energy, by 30 and 50% as well as 29 to 30%. As machine flow rate dropped, these ones were increasingly noticeable and were shown closely connected to machine features. It’s also important to note that machine’s performance declined since the increased tip clearance merely made dissipation of turbulent for tip blade region worse. Using the aforementioned entropy theory, Yang et al.^12^ examined losses pump energy in different mode of operating with identified the most significant regions of flow. Al-Obaidi and Alhamid^13^ pointed out that dissipation of energy could occur around 50% in impeller itself because of phenomena of complex flow such as back flow in inter-blade region at arose vortices as separations of flow in trailing and leading blade edges. These investigations have once again come to conclusion that volute and impeller casing regions of flow establish flow for complex losses in pumps. Cai et al.^14^ used optimisation strategy to try to enhance the pump performance with changing load circumstances. For the aforementioned operating conditions, they were able to increase efficiency of machine through 20%. Improved model’s inter-blade flow field then reached a much superior condition, where, for example, under part-load, the pressure in impeller blade variations were unchanging with significantly reduced in vortices. An attempt was made in another research^15^ to optimize the pump and turbine modes for design circumstances at a single speed, as well the outcomes were satisfactory. Nguyen et al.^15^ carried out a special designed of impeller with blades have curved type forward for use to pump after realizing that this pump design has separations in flow in edge pf blade leading are nearly inevitable. The higher BEP flow conditions were caused by the increased blade inlet angle. New design demonstrated a 14% improvement in efficiency of machine when compared to standard version. While Fei et al.^17^ examined the impact of rotation speed, Savar et al.^16^ examined the clocking effect on pump performance while taking into account a diffusor type of pump. Zhou et al.^18^ examined the same parameter, but this study examines its impact on tip leakage vortex while taking into account mixed pump type. It was usually discovered that rise in rotation speed exacerbates tip leaking vortex. Regarding mixed and axial pumps, it was less well-known varieties area, some findings were released with a primary emphasis on tip leaking vortex dynamics^19,21^.

One must examine the features of the pump as well impact of many limitations on it in order to get a comprehensive grasp of pump mode operations and related flow dynamics, particularly with regard to pump flow. With a primary focus on the pump’s pumping mode, the current research offers a numerical analysis that explores the method of local flow structure construction and how it eventually evolves when machine flow circumstances shift from part-load to full-load. Furthermore, three blade angles −3°, 0°, and 3° were taken into consideration in an effort to examine the mechanism by which the impeller blade angle affects local flow dynamics and related pressure pulsation features. In order to identify the distribution patterns of flow, this study examines the intricate flow structures inside the numerous hydraulic components of the axial-flow model under various operating situations. The investigation findings can attend as guide for future axial-flow optimization.

## Physical model and numerical approach for pumps

### Geometric model of a pump

**Fig. 1:**
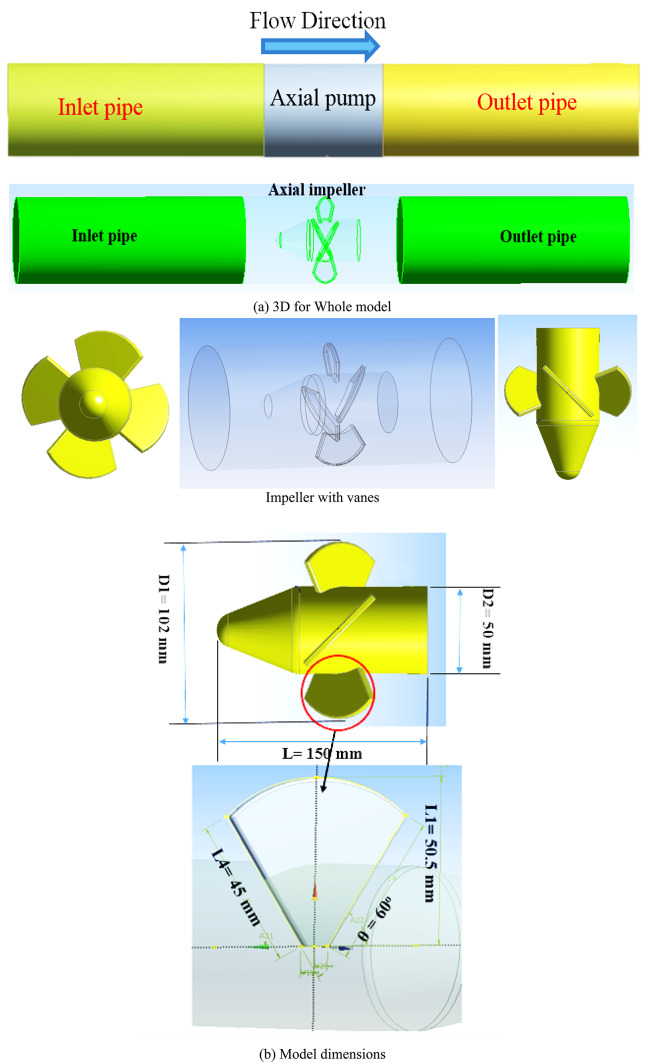
Pump components and computational domain.

The four primary parts of the axial pump model type under investigation in this study are the intake pipe, impeller, diffuser, and output pipe. It has a high speed (N = 3000 rpm). Table 1 displays model geometrical parameters. There are 4 impeller blades. The analyzed computational domain, which includes the four components indicated above, was constructed for numerical simulations using the Fluent CFD technique, a geometrical modeling program. The components and computational domain used are displayed in Fig. 1. The computational domain at the inlet and exit pipes is enlarged, as shown in Fig. 1, to avoid the impact of boundary condition constraints, guarantee that flow within intake pipe was completely developed, as well produce computational stability findings. This study’s axial pump design flow characteristics include an impeller speed 3000 rpm, four impeller blades, as well as design flow was 12.5 L/min. Fluent (CFD method) was used to develop a numerical pump model in three dimensions. Figure 1 shows 3D representations of the entire model, including the guide vane, axial impeller, intake, and exit. The flow conditions in this investigation were including five conditions 5, 10, 12.5), 17.5, and 20 l/min respectively.

**Table 1:** Details test of axial pump.

Portion	Unit and value
Speed design	3000 rpm
Head design	3 m
Hub diameter	50 mm
Flow design	12.5 l/min
Tip diameter	102 mm
Blade thickness	2 mm
Number of blades	4
Pump speed	3000 rpm
Blade angles	60^o^
Power design motor	2.4 kW
Ratio tip to hub	0.5

Numerical simulations are conducted in this regard, taking into account water that enters computational domains including intake region of inlet pipe, travels by inter-blade flow channels of the impeller, and finally exits the domain through the outlet pipe. This trajectory is modeled after an experimental procedure in which the flow was directed in the same manner. In contrast to several previously published publications, the model under investigation does not provide any clearance at the tip of the impeller blade. Every experiment was carried out in accordance with international guidelines for evaluating hydraulic apparatus. The machine’s exterior characteristics of performance were experimentally tested in a variety of flow situations, including varying flow conditions.

**Fig. 2:**
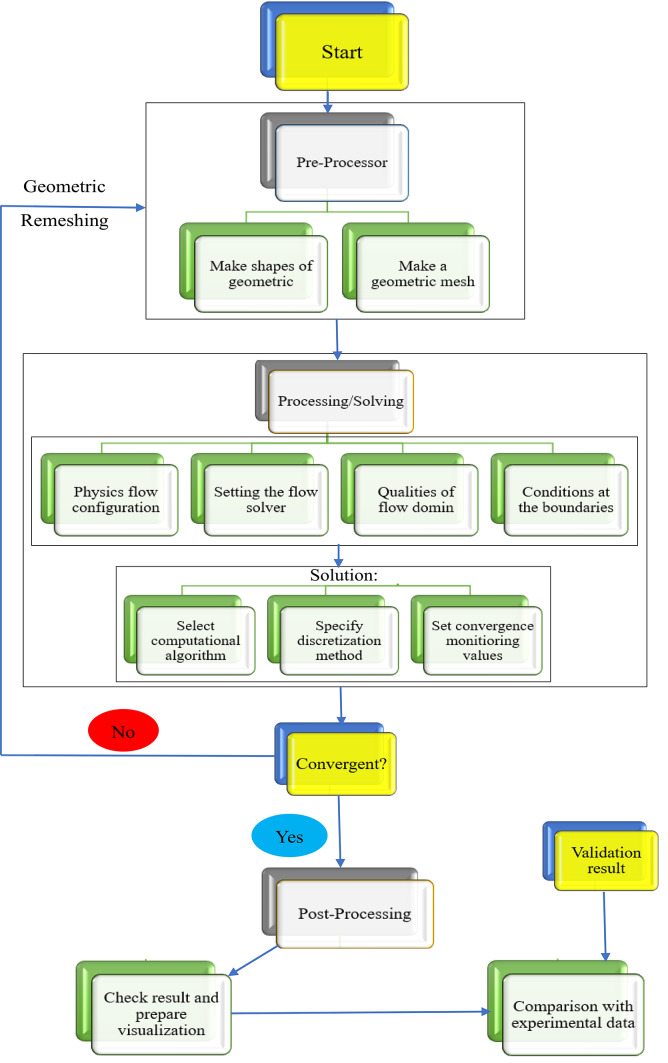
The CFD research’ numerical methodology.

This study examines how pump affect under different transient conditions with dynamic simulation that were enhanced above pump. It created 3D models of different part of pipes and pump with axial impeller using a ANSYS Fluent 2022R1. Thus, the study starts in this part by looking at a physical model and how its general form (configuration) is influenced by its shape (geometry). The system’s governing mathematical formulas and crucial information on the computer simulations are then provided. The techniques employed to guarantee the simulations’ accuracy (grid independence) are next covered. Lastly, the correctness of the simulations is confirmed by comparing the findings with real tests conducted on both smooth and textured (dimpled) tube forms. Three sections of the CFD procedures are detailed in Fig. 2.

### Generation of computational grids

**Fig. 3:**
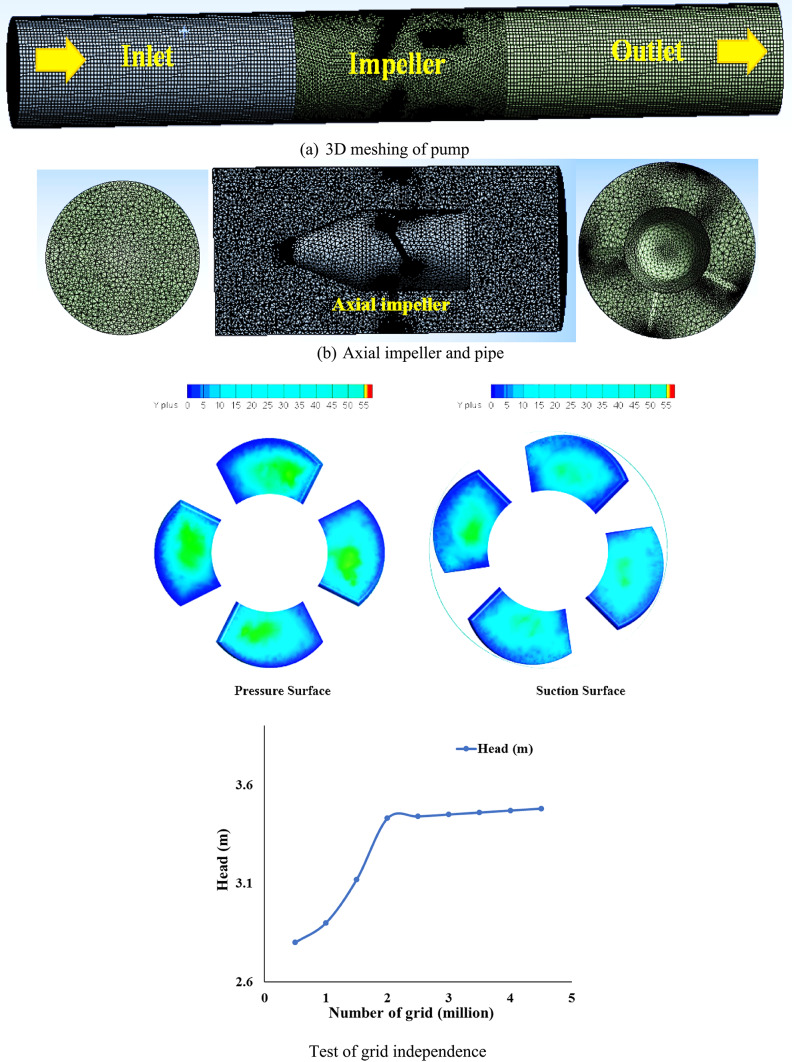
Grid selection for the input pipe (**a**), impeller (**b**), and outlet pipe (**c**) of the various model components.

According to the primary goals of this study, numerical simulations are carried out to look at machine dynamics flow process of development as well that causes the various performance traits that are displayed in the sections above. A computational grid must be created from the whole computational area of concern as a first step as illustrated in Fig. 3 and Table 2. The computational grids of the four components of the pump model were generated in this work using the ANSYS ICEM software, with the hexahedral scheme being employed for the majority of the flow domains. Maximum y + was a reduced amount of than 15, a relatively tiny mesh was created at regions of flow of considerable position, for example impeller blades. Entire mesh domain includes inlet pipe, axial impeller, and exit section. Because of the intricate geometry of the axial pump and the structure of complex computational field in internal flow, unstructured meshing (tetrahedral mesh) is utilized. The ANSYS ICEM mesh was applied for interface between impeller and local refinement was used for important flow field areas.

**Table 2:** Particular grid data for each pump element.

Components	Pipe at inlet	Impeller	Pipe at outlet
Type of grid	Type of hexahedral	Type of tetrahedral	Type of hexahedral
Number of grids (Million)	1.4	3.2	1.1
Quality of grid	3.3	2.7	3.3

Different distinct number of grids were between 0.5 to 7.5 million evaluated in a grid independence test in order to rule out potential that the grid number being used may impacted the outcomes of numerical simulations. Each grid set has undergone steady state numerical simulations with comparable boundary conditions. The resulting characteristics performance foe pump were then compared. It was below 6.5 million as seen in the figure, but for grid numbers above that, it has nearly stabilized. Therefore, grid of 5.5 million was selected for additional simulations in accordance with the computing resources available. While Figure provides a brief overview of the created mesh at all flow regions, Table 3 displays the specifics of the chosen grid for various pump model components.

**Table 3:** The primary apparatus used in the experimental test configuration.

Parameter	Part of device	Error systematic
Pump head	Inlet and outlet pressure transducers	± 0.14% ± 0.14%
Sensor of vibration	Sensor of accelerometer	± 0.19%
Torque and speed	Sensor of speed torque	± 0.18%
Water flow	Flow meter	± 0.16%

The incompressible fluid motion equations.

1 $$\frac{{\partial \rho_{m} }}{\partial t} + \frac{{\partial (\rho_{m} u_{j} )}}{{\partial x_{j} }} = 0$$

2 $$\frac{{\partial (\rho_{m} u_{j} )}}{\partial t} + \frac{{\partial (\rho_{m} u_{i} u_{j} )}}{{\partial x_{j} }} = - \frac{\partial \rho }{{\partial x_{i} }} + \frac{\partial }{{\partial x_{j} }} \left[ {(\mu_{m} + \mu_{t} )(\frac{{\partial u_{i} }}{{\partial x_{j} }} + \frac{{\partial u_{j} }}{{\partial x_{i} }} - \frac{2}{3}\frac{{\partial u_{k} }}{{\partial x_{k} }}\delta_{ij} } \right]$$

Equation provides the related equations for kinematic eddy viscosity, specific dissipation rate, and turbulent kinetic energy.

3 $$\frac{{\partial \left( {\rho_{m} k} \right)}}{\partial t} + \frac{{\partial \left( {\rho_{m} k} \right)}}{{\partial x_{j} }} = P_{k} - D_{k} + \frac{\partial }{{\partial x_{j} }} \left( {(\mu_{m} + \frac{{\mu_{t} }}{\sigma k}} \right)\frac{\partial k}{{\partial x_{j} }})$$

4 $$\frac{{\partial \left( {\rho_{m} \omega } \right)}}{\partial t} + \frac{{\partial \left( {\rho_{m} u_{j} \omega } \right)}}{{\partial x_{j} }} = C_{\omega } P_{\omega } - \beta_{\omega } \rho_{\omega } \omega^{2} + \frac{\partial }{{\partial x_{j} }}\left( {(\mu_{m} + \frac{{\mu_{t} }}{\sigma k}} \right)\frac{\partial \omega }{{\partial x_{j} }})2\rho_{\omega } \left( {1 - F_{1} } \right) \sigma \omega^{2} \frac{1}{\omega }\frac{\partial k}{{\partial x_{j} }}\frac{\partial \omega }{{\partial x_{j} }}.$$

The viscosity is

5 $$V_{t} = \frac{{\rho a_{1} k}}{{\max \left( {a_{1} \omega ,SF_{2} } \right)}}$$

where ρ, k, μ, u and P the density, thermal conductivity viscosity and pressure of fluid.

### Experiential test

**Fig. 4:**
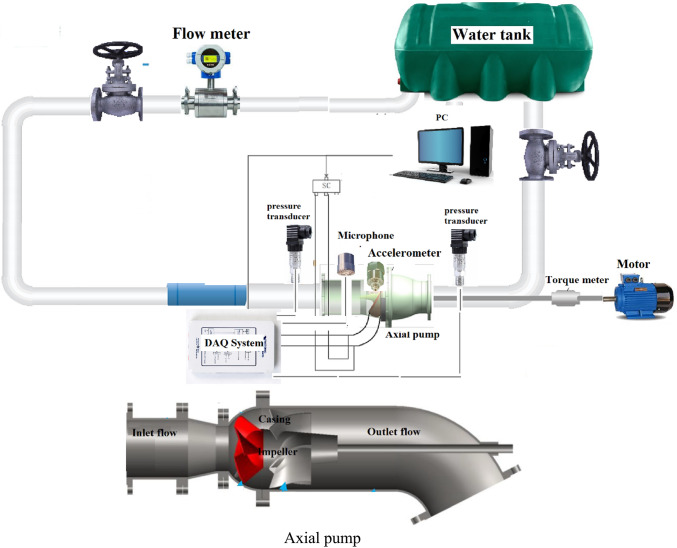
Components and experimental test rig.

On the test experimental bench, there are two additional control valves: one upstream and one downstream of the test valve as illustrated in Fig. 4. A control system regulates these two valves as well as the pumps, changing the volumetric flow over the test bench as a whole. With the two additional valves, the pressure around the test pump may be changed both upstream and downstream. All of the sensors and the test valve are positioned between these two external control valves. The pipes connecting the test pump and the external valves are long enough to allow for continuous flow for precise readings. To keep the vapor pressure almost constant and to avoid temperature influencing cavitation, the water temperature was maintained between 23 and 30 ◦C. The pump’s body has a single piezo element installed for the immediate purpose of capturing the noise the construction produced.

### Uncertainty in experiments

Figure 4 is a drawing of the experimental pump equipment. The length and straightness of the pipe section in front of the electromagnetic flow meter ensured a smooth flow into the device and test accuracy. The flow meter was used to measure flow and was examined before installation. The differential pressure transmitter speed torque sensor kept pump shaft and motor apart. It monitored input torque as well pump shaft speed to determine head. Head and efficiency error were the square root of random error [21].

6 $$E\eta = \pm \sqrt {E_{\eta ,s}^{2} + E_{\eta ,R}^{2} }$$

Sum of square roots is used to determine on test bench.

7 $$E\eta ,s = \pm \sqrt {E_{Q,s}^{2} + E_{H,s}^{2} + E_{n,s}^{2} + E_{M,s}^{2} }$$

where $E\eta ,s$ is the electromagnetic flowmeter, E_H,S_ is the differential pressure transmitter, E_M,S_ is the torque meter, and E_n,S_ is the torque speed sensor’s systematic error. Table 3 lists the testing equipment required to determine these systematic error characteristics.

**Fig. 5:**
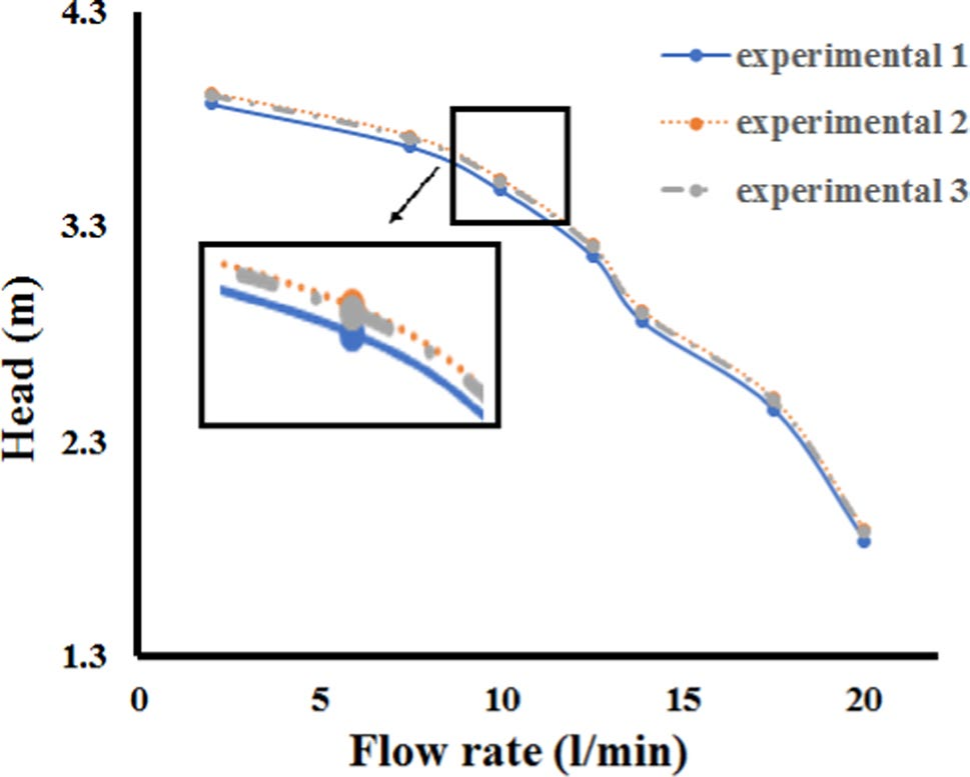
Verify the pump’s repeatability.

Test outcomes were revealed in Fig. 5. Flow-head curves of three tests of an axial pump exhibit patterns that are almost the same as the flows increase. The test findings appear to be trustworthy because the data collected under similar operating conditions is similar.

**Fig. 6:**
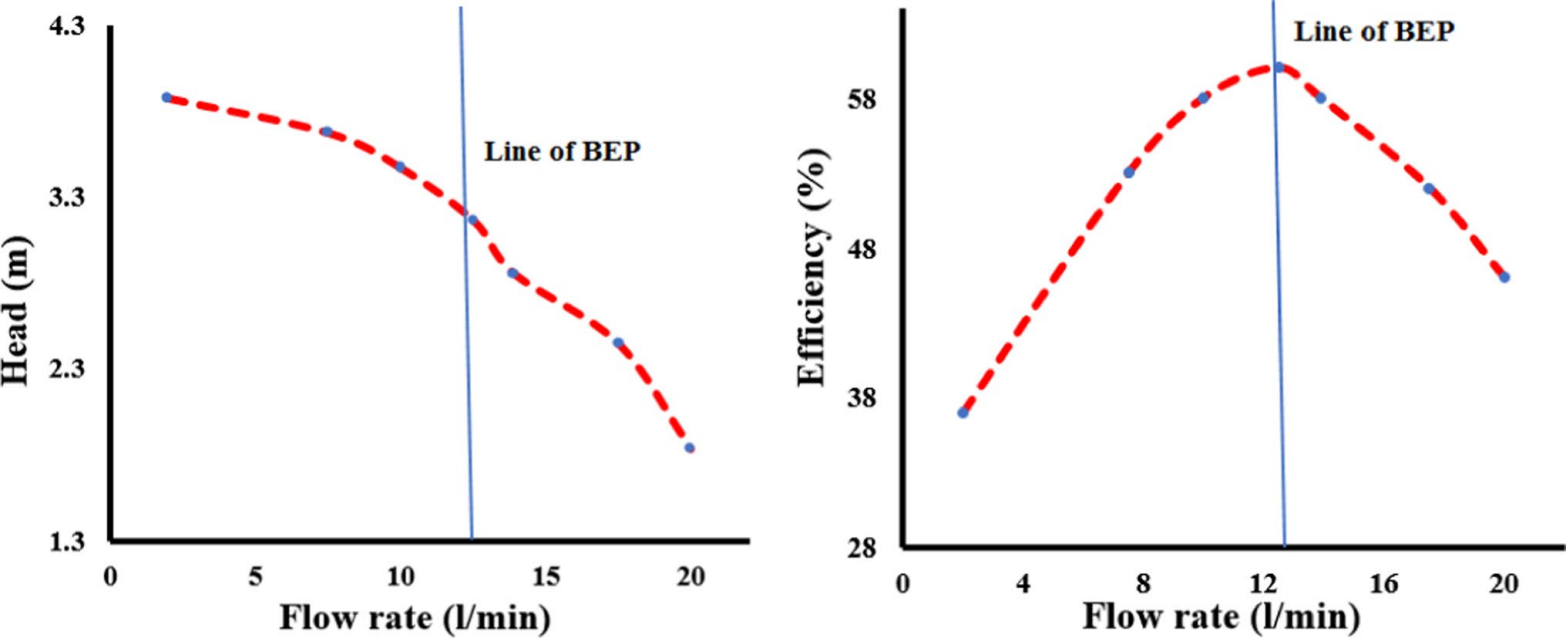
External performance parameters of the machine.

The experimentally determined characteristics of external performance foe head as well pump efficiency were presented in Fig. 6, which also displays rig of experimental. BEP line is vertical line in graph, and view schematic for testing is revealed in this figure, taking into account the impeller blade angle of -3°. It can be observed that while efficiency rose to BEP point before declining for further flow circumstances, both the produced head fell with a continuous flow discharge increase. The optimal efficiency was attained under 12.5 l/min flow circumstances after conducting experimental experiments on a variety of flows.

### Scheme for numerical simulation and validation

The commercial CFD code Ansys CFD approach was used to do 3D simulations of pump flow with several circumstances. This code solves RANS equations of motion of incompressible fluids, applying turbulence model type Shear Stress Transport.

Since system under investigation employs water with temperature 25°C as well hydraulic pumps are used for both pumping and energy generation, it was as incompressible fluid classified. Equations 1 and 2 present the Cartesian version of RANS and continuity equations. Viscosity fluid pressure, body forces and density were represented by variables μ, p, Fi and ρ in these formulas. Turbulence model type of SST k-ω, which was created by Menter [30–32], combines benefits of two earlier as well widely used models for k-ε and k-ω. Improves the ability to predict flow dynamics both in the fluid bulk far from the wall and within the wall vicinities. Additionally, this model has a reputation for performing well, particularly in simulation scenarios including opposed gradients of pressure as well as separations in flow [34]. Equations 8, 9, 10 provide the corresponding equations for viscosity of kinematic eddy, dissipation rate, and turbulent kinetic energy.

8 $$H = \frac{P2 - P1}{{\rho g}}$$

9 $$P = \frac{2\pi nT}{{60}}$$

10 $$\eta = \frac{\rho gQH}{P} X 100$$

where H, P and ƞ the head, power and efficiency of pump.

**Fig. 7:**
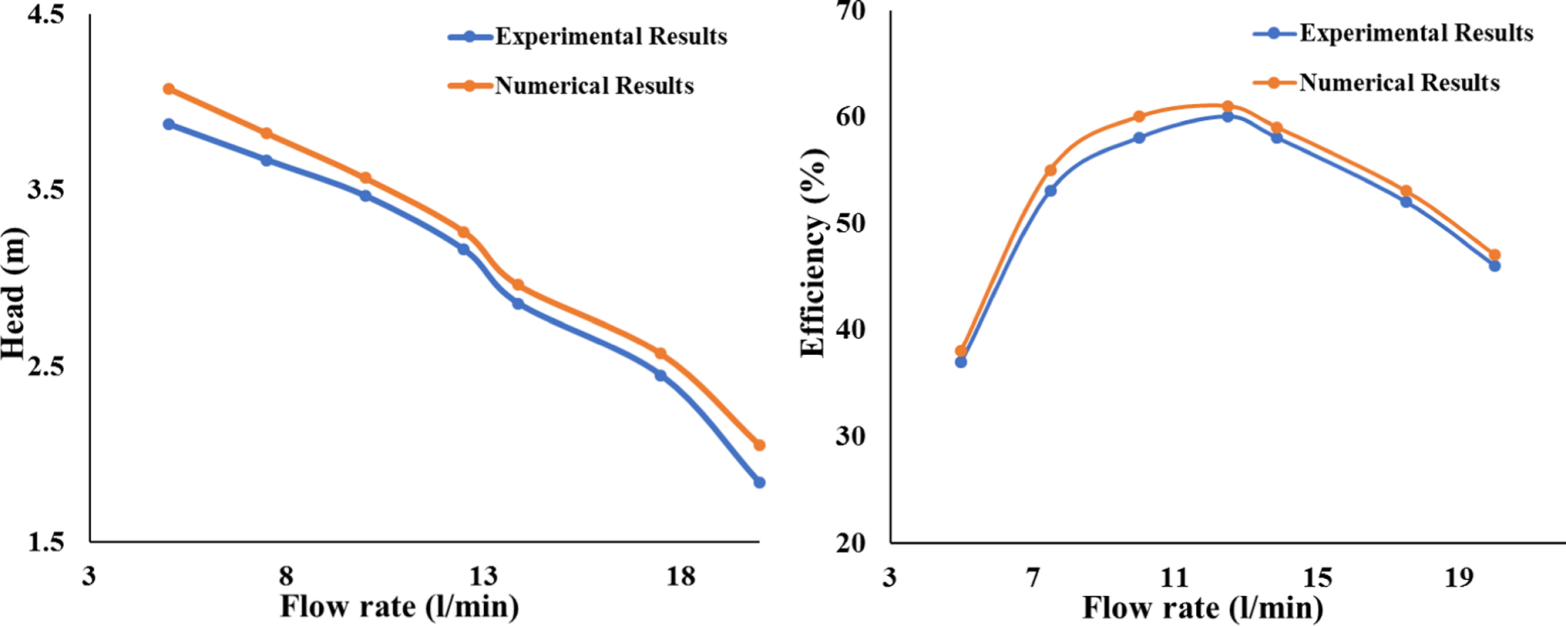
Comparison of experimental and numerical findings.

Outcomes of numerical simulations were used as beginning conditions for transient numerical simulations during 3D of pump flow with several situations, with the goal of achieving a rapid and solution convergence as illustrated in Fig. 7. The values of the used boundary conditions for each simulation session were selected from the experimental data that was previously displayed. The current analysis only considers a single blade angle θ = -3° and a limited number of flow circumstances, despite the fact that experimental testing was carried out on flows as well blade angles (θ). Four scenarios were taken into consideration for varying circumstances. However, taking into account the most unstable flow situations, the impeller blade angle was raised for two consecutive values, θ = −3°, 0° and + 3°, to examine impact of blade design on pump flow instability. Entire pump was split into fixed and rotating during the simulation process, and various interfaces were employed in between. For example, for transient rotor–stator was carried out in same flow regions.

The general grid interface was used for different interfaces connecting fixed components. Condition of non-slip wall was applied to every other wall in relevant simulation, furthermore to P_in_ and Q_out_ serving as inlet velocity and pressure outlet boundary requirements. For both turbulence numbers and the advection method, a high-resolution scheme was used. One complete impeller rotation is equal to 3600∆t, as the timestep ∆t for transient flow solution was specified as amount of time needed for the impeller to revolve one degree. Additionally, for every timestep, five internal loops were chosen. The convergence criteria for each solution was defined as RMS residuals lower 10^–5^, and each solution was made to run for ten impeller rotations. As illustrated in Figure, to verify the accuracy of the numerical outcomes produced using the numerical simulation scheme previously presented. To achieve this, final parametric values were calculated by averaging numerically determined variations of each of varying parameters. In other words, numerical findings derived using this numerical method may be adequately compared to real-life situations since the global error data was less than 5%. It should be noted that the formulae used for Head, Power, and Efficiency are displayed in Eqs. 8, 9, and 10.

## Results and discussion

### Vibration analysis

**Fig. 8:**
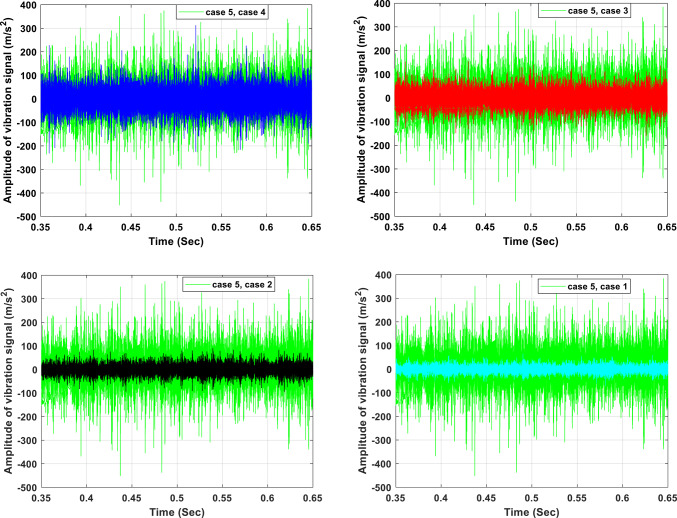
Comparison of vibration signals in different flow conditions in time investigation.

Sample portions of the axial pump’s vibration waveform recordings are displayed in Fig. 8. Five examples with different flow conditions were used to measure the records. The waveforms recorded at the accelerometer’s axial positions were shown in this image. It is observed that the vibration signals are less in amplitude and mostly consist of lower frequency components, and the pump is typically quieter at lower flow conditions. Moreover, as flow conditions increase the vibration signals are higher in amplitude as shown condition 5 conditions. The original time domain signals under various situations are shown in this figure. It is possible to see the variations in time-domain vibration signals under various circumstances using this time-domain signal. Additionally, there are oscillations in the time-domain vibration signals that allow the information contained in the vibration signals to be seen.

**Fig. 9:**
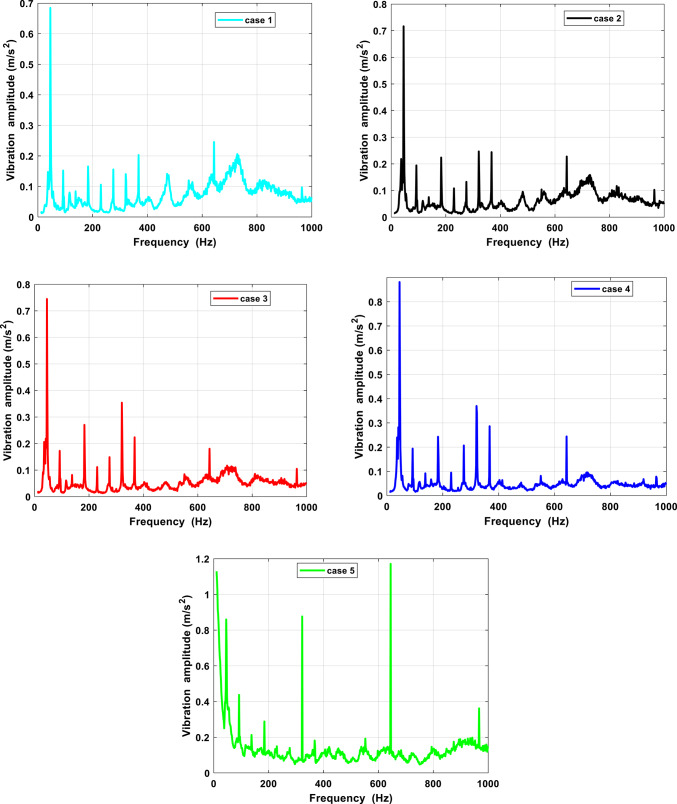
Comparison of vibration signals in different flow conditions in frequency investigation.

The frequency-domain analysis of the pump vibration signal presented in Fig. 9 indicates that the majority of vibrational energy resides in the frequency range below 1 kHz. This distribution aligns well with earlier experimental and numerical investigations of axial pumps, where vibration is predominantly governed by hydraulic excitation and rotor–stator interaction mechanisms. Frequencies within this band are typically associated with pressure oscillations, blade-related excitation, and structural resonance effects, rather than high-frequency phenomena such as bearing degradation or electrical disturbances. Comparable spectral characteristics have been reported for pumps experiencing unsteady interactions between impeller blades and the volute tongue, in which fluctuating pressure fields constitute the principal excitation source. The spectral analysis reveals that the most prominent peaks correspond to the shaft rotational frequency and its multiples, with the blade passing frequency (BPF) consistently emerging as the dominant excitation component across most operating regimes. At the nominal rotational speed of 3000 rpm, the pronounced BPF component confirms that the observed vibration response is largely induced by periodic hydraulic loading arising from blade–volute interaction. This interpretation is strongly supported by existing studies on pump vibration and acoustic behavior. Furthermore, the close similarity in spectral content measured at different axial sensor positions suggests that the vibration response reflects a system-wide hydraulic excitation rather than localized mechanical faults or structural defects.

From a mechanistic perspective, the coupling between pressure fluctuations and vibration can be explained by the transmission of unsteady pressure loads generated within the impeller flow passages to the pump casing and shaft assembly. These fluctuating forces excite structural modes whose natural frequencies lie within the same spectral range, leading to resonance amplification. In the presence of flow instabilities or incipient damage, the magnitude and temporal characteristics of these hydraulic forces are altered, resulting in enhanced vibration amplitudes while maintaining the same dominant excitation frequencies. This behavior explains why off-design operation or fault development increases vibration intensity without introducing new characteristic frequencies. Operating flow rate plays a critical role in shaping the vibration spectrum. Under high-flow conditions, vibration levels increase significantly, with amplitudes reaching approximately 3.5 times the BPF and a peak acceleration of about 1.1 m/s^2^. Such amplification has been widely attributed in the literature to intensified flow separation near the impeller outlet, stronger rotor–stator interaction, and elevated turbulence levels. Although vibration amplitudes are reduced at the design flow rate, the continued presence of BPF harmonics within the 0–0.6 and 0–0.8 frequency ranges demonstrates that blade-induced pressure pulsations remain the dominant excitation mechanism even during optimal operation.

Conversely, at low-flow operation, the spectral energy shifts toward higher-order harmonics, particularly around 4BPF, accompanied by a notable increase in vibration amplitude. This shift is physically associated with severe flow non-uniformities, including inlet backflow, rotating stall, and pronounced recirculation zones, which impose highly uneven pressure loading on the impeller blades. Experimental studies on axial pumps have consistently identified the dominance of higher BPF harmonics under part-load conditions as a clear indicator of hydraulic instability. As the flow rate increases toward overload operation, the BPF once again becomes the prevailing frequency component, while higher-frequency content diminishes, reflecting a transition toward more periodic and blade-synchronous excitation. Nevertheless, at extreme high-flow conditions, the overall vibration magnitude rises again due to the formation of large-scale vortices and unsteady wake interactions, which intensify pressure fluctuations and amplify the vibration response. The presence of multiple spectral peaks and their systematic variation with flow rate confirms that pump vibration behavior cannot be attributed solely to mechanical imbalance. Instead, it is predominantly governed by hydrodynamic excitation that is strongly modulated by operating conditions. Although the rigid connections in the experimental setup may transmit secondary vibration modes induced by fluctuating hydraulic forces, their contribution remains minor compared to blade-passing excitation. Overall, the findings demonstrate that while the number of impeller blades establishes the fundamental excitation frequency through the BPF, the operating regime dictates vibration amplitude and harmonic structure via pressure fluctuation intensity and vortex dynamics.

**Fig. 10:**
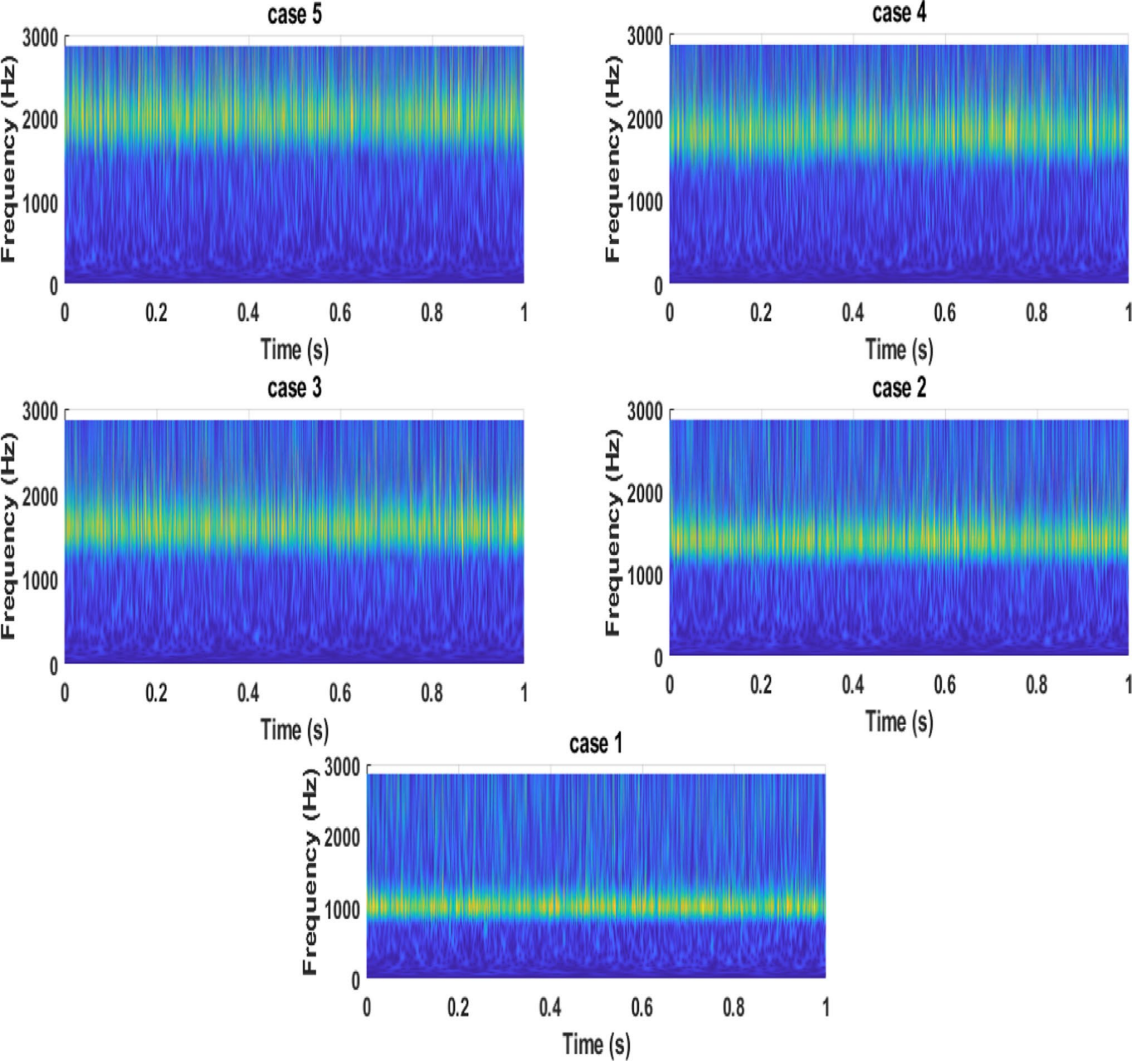
Comparison of vibration signals in different flow conditions in frequency using CWT investigation.

To characterize the nonstationary nature of the vibration signals, the continuous wavelet transform spectrum (CWTS) was utilized to examine their time–frequency behavior. The resulting CWTS representations under different operating regimes are illustrated in Fig. 10, where the evolution of vibration energy across both time and frequency domains can be clearly observed. Unlike conventional FFT-based analysis, which assumes signal stationarity, the CWTS enables identification of transient excitation processes associated with unsteady hydraulic activity within the pump. The time–frequency maps display distinct signatures for each flow condition, demonstrating that the vibration response is highly sensitive to changes in operating regime. At elevated flow rates, vibration energy becomes strongly concentrated within higher frequency bands. This behavior is consistent with intensified pressure pulsations arising from enhanced rotor–stator interaction and increased turbulence near the impeller discharge region. Comparable concentration of high-frequency vibration energy under overload operation has been documented in earlier experimental studies, where vigorous wake interaction and flow separation amplify time-dependent hydraulic forces.

Under nominal and moderate flow conditions, the CWTS patterns exhibit a more uniform temporal distribution of energy, with dominant components aligned with blade-related frequency ranges. This response indicates relatively stable and periodic pressure oscillations synchronized with blade-passing events, suggesting that blade–volute interaction governs the vibration behavior in this operating window. The reduced intermittency observed in the time–frequency domain further implies a weaker excitation of structural resonance compared with off-design conditions. In contrast, operation at low flow rates produces sporadic, broadband energy clusters extending across multiple frequency scales in the CWTS representations. These features are indicative of severe hydraulic instability, including inlet recirculation, rotating stall, and backflow, which generate irregular and non-periodic pressure fluctuations. Such unstable flow structures impose highly intermittent hydraulic loading on the impeller blades, resulting in localized bursts of vibration energy that are effectively captured through wavelet-based analysis, as reported in previous investigations. Taken together, the CWTS results demonstrate a clear physical linkage between vibration response and pressure fluctuation dynamics. As the pump transitions from part-load to overload operation, the vibration energy distribution evolves from dispersed, broadband patterns to more focused high-frequency bands driven by intensified hydraulic excitation. This progression confirms that pump vibration is predominantly controlled by flow-induced pressure fluctuations rather than purely mechanical sources and highlights the effectiveness of CWTS as a diagnostic approach for identifying flow-dependent vibration mechanisms in axial pumps.

### Numerical features of the flow field investigation

**Fig. 11:**
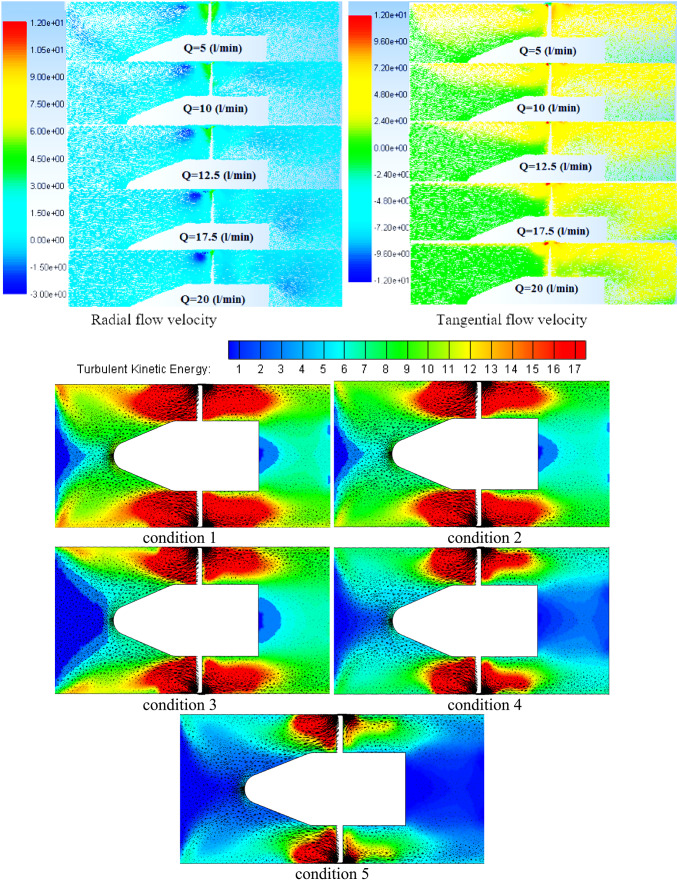
Impeller blade flow region’s contours for flow radial, tangential velocities and TKE under various conditions flow conditions.

Different planes inside impeller blade flow were taken into consideration in order to examine the potential variations in dynamics flow with impeller under various flow circumstances as illustrated in Fig. 11. These planes grow from hub region to shroud. These planes take up sequential positions, while taking into account unitary spanwise between impeller hub and shroud. The figure displays the impeller flow radial, tangential velocities and turbulent kinetic energy (TKE) distribution contours and the associated vorticity measure under various flow conditions. As the machine inflow is gradually increased from low conditions to BEP conditions, the flow regions closest to the hub have the lowest flow speeds, while flow regions closest toimpeller shroud have the greatest as shown in TKE in this figure. Furthermore, these velocities contours demonstrate that both the impeller blade’s suction side and the impeller outlet region record greater velocity flow on specific planes. Additionally, it is evident that the blade suction side’s high speed flow regions enlarge as they move spanwise toward shroud as well extend farther in a streamwise way into downstream regions.

Furthermore, at higher BEP conditions, the increased machine influx has caused a constant expansion of the flow regions employed through higher velocity on side of blade suction. These flows reach to edge of blade trailing and begin to expand towards mid-region of blade passages before touching the trailing region of blade pressure region. The flow separation that occurred at leading of impeller blade edge as well as blade region vicinities in downstream flow was thought to be the source of this characteristic. Under deep part-load flow circumstances, inlet swirling flow intensifies separation in flow at blade region in downstream regions, causing it to clearly separate from the blade leading edge one in the vicinity of the impeller hub. Though, it is discovered that this detachment weakens in the spanwise direction, causing both separation in flow region to rejoin and create a single, enormous region that extends the whole blade length at flow regions near the impeller shroud. Under deep part-load circumstances, it is also discovered that a considerable flow incidence angle is associated with flow separation at the blade leading edge. It was discovered that when the machine flow discharge decreases, the flow incidence angle at the leading edge of the impeller blade progressively rises, ultimately causing the formation of massive flow wakes at the same region, particularly in the hub and shroud vicinities of the impeller blade under low flow conditions.

In a similar vein, the velocity swirling strength parameter, which is used to describe the flow vorticity in impeller blade region, has not only validated the previously mentioned elements but also indicated the existence of two additional high vorticity regions. One is fastened to the pressure side of the impeller blade near the mid-length of the impeller, while the other is attached to the trailing edge of the blade. The latter extends in the opposite direction of the impeller ’s rotation, swiping the blade trailing edge region in the shape of a wake structure and clearly occupying the high speed flow region mentioned above within the impeller outlet vicinities. It naturally connects with the vaneless space vortex flow before meeting the pressure side vortex of the next blade. Additionally, it is discovered that the high vorticity region at the impeller outlet progressively contracts in a spanwise direction until it reaches the mid-span region, after which it expands once again in the direction of the shroud vicinities. At this point, wake vortices attached to the blade’s trailing edge are too short to reach the pressure side-associated vortex of the following blade. Rather, the screw-like vortex appears at the impeller suction side and extends from the shroud tip area of the blade leading edge to the downstream region, where it progressively grows larger towards the impeller outlet region. This structure, which is connected to the impeller shroud, deteriorates as the machine flow discharge increases, just like any other secondary flow that was explained.

**Fig. 12:**
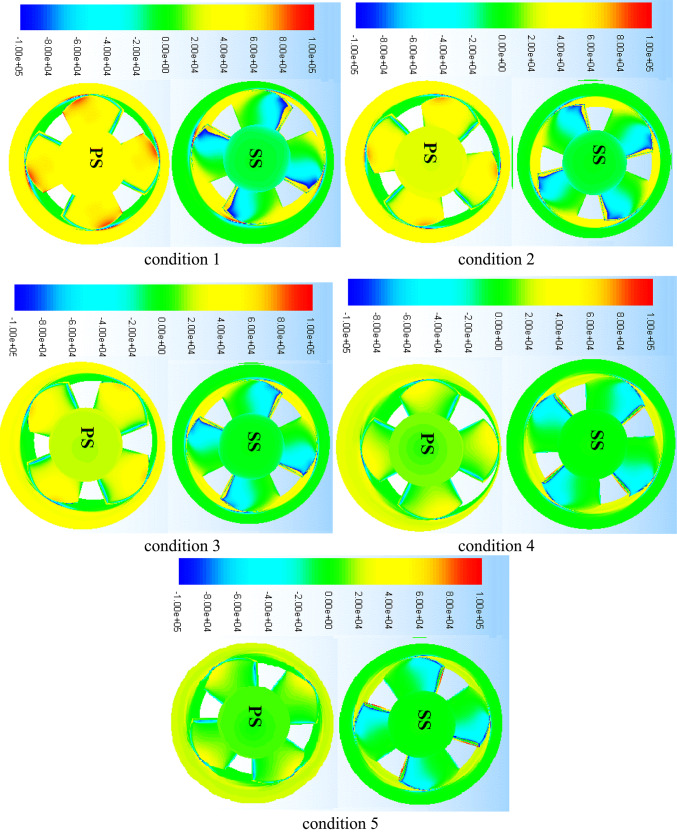
Pressure contours on regions of suction and pressure of blade under conditions flow circumstances.

A graphic representation of the local pressure distribution mode on the suction and pressure surfaces of the impeller blade was included in Fig. 12 to help clarify the previously stated issues. Beginning with the blade suction surface, the local pressure distribution mode may be clearly divided into two regions: the high pressure region at the trailing edge and vicinities of the impeller blade and the low pressure region at the leading edge and vicinities of the blade. But from the blade leading edge downstream, there is a seamless transition between regions, and the size of each region changes according to the operating circumstances taken into account. The low pressure region at the blade leading edge continues downstream to a certain blade mid-length distance and covers the whole spanwise blade length from hub to shroud, which is in good agreement with the velocity distribution mode previously stated. Furthermore, compared to the hub region, this region is longer in the shroud region. In the sections above, it was demonstrated that high velocity regions are narrow in the hub region but develop downstream in the layers above, such as the flow regions close or inside the shroud vicinities. This is in good agreement with the flow velocity distribution pattern on the blade suction side. As the machine flow discharge increases, it is also observed that these low pressure regions around the shroud expand farther downstream. A corresponding high pressure sector inside the hub vicinities is observed to increase in size with the machine flow discharge, demonstrating the above-discussed separation of high velocity regions on the blade suction side from the effect of the inflow swirling flow in the hub region. However, the circumstances on the blade’s pressure surface are the reverse of those on the suction surface.

High pressure regions on this side are concentrated in the vicinal region of the blade’s trailing edge and expand from the hub to the shroud region. Furthermore, it is discovered that when machine flow discharge increases, the size of the high pressure region in the shroud vicinities decreases. This is consistent with the flow vorticity distribution mode and related evolution in the same regions that were previously studied.

**Fig. 13:**
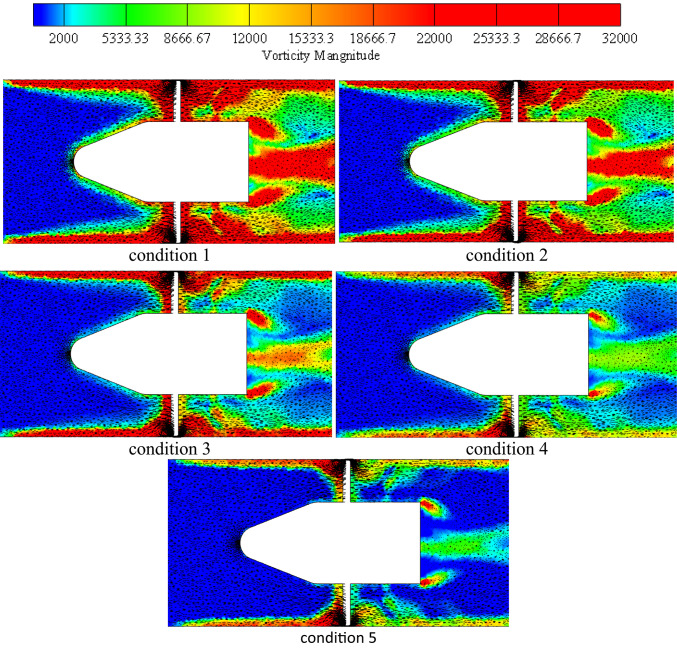
Impeller’s primary flow vorticity regions at various flow conditions.

Velocity-colored flow streamlines from the impeller intake to the outlet on a transversal plane are shown in Fig. 13 for different consecutive instants. In this Figure, represents the beginning time, and T is the impeller rotational period. This graphic illustrates the instantaneous variations in impeller flow topologies under different load operation scenarios. It was demonstrated worldwide that when machine flow discharge decreases, impeller flow vorticity increases and becomes more robust. Furthermore, despite the shift in machine flow conditions, the placements of the principal flow vortical structures appear to stay the same. They like to become bigger or smaller. The impeller outlet vortex, which is mostly located inside the vicinal regions of the impeller hub, as well as the blade pressure side and blade suction side -attached vortices and their expansions in shroud vicinities away from the blade, are seen in this image. For deep part-load circumstances, the latter is seen to enlarge and spread to the mid-span region and beyond. According to their placements, the impeller flow vorticity may be divided into different parts globally, as shown in the figure: the blade leading edge vortex, blade suction side vortex, blade pressure side vortex, and impeller outlet area vortex. The principal vortex flow sites in the impeller flow region for the different load circumstances under investigation are shown in the figure. As a result, the figure’s middle and right sections display the impeller blade suction side and blade pressure side with the appropriate flow vortices, while the left portion displays the impeller assembly with the primary flow vortex locations marked. In perfect accord with the aforementioned parts, the blade leading edge vortex expands spanwise until it reaches the impeller shroud region, from whence the blade suction side vortex originates. From there, it extends the whole blade length and beyond, mostly remaining inside the shroud vicinal region. This structure may connect with the impeller outlet area vortex at the impeller outlet and extend in the opposite direction of the impeller rotation to finally connect with the blade pressure side vortex of the preceding blade, depending on the machine flow parameters taken into consideration. As shown in Figure, this is the condition for the deep part-load scenario. The blade pressure side vortex does the same when shifting places with varying flow circumstances, but all of these vortices are observed to decrease and finally disappear as the machine flow conditions change from part-load to rated operating conditions. The blade pressure side vortex stretches from the mid-length of the blade downstream at deep part-load circumstances (condition 1). When the flow reaches part-load conditions (condition 2), it descends spanwise to the mid-span region and now forms a string-like extension from blade leading edge to the impeller outlet.

**Fig. 14:**
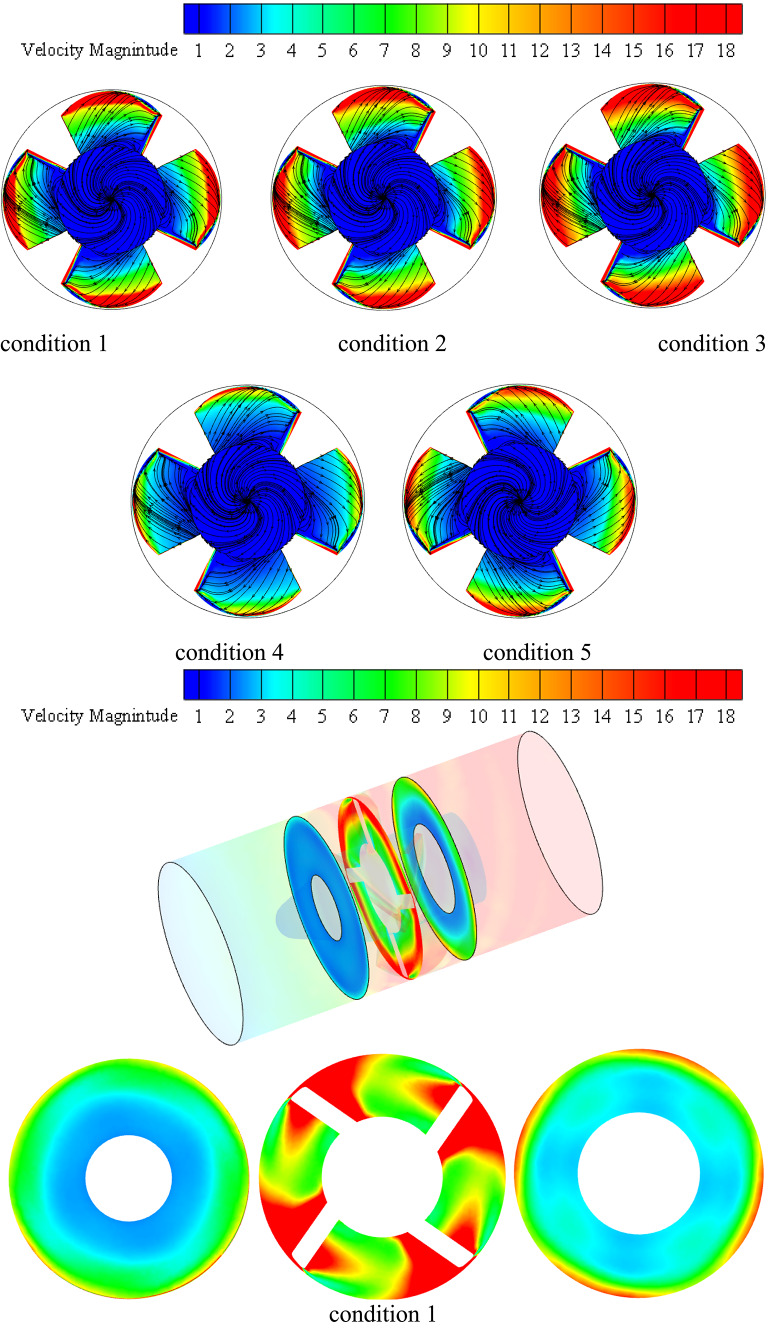

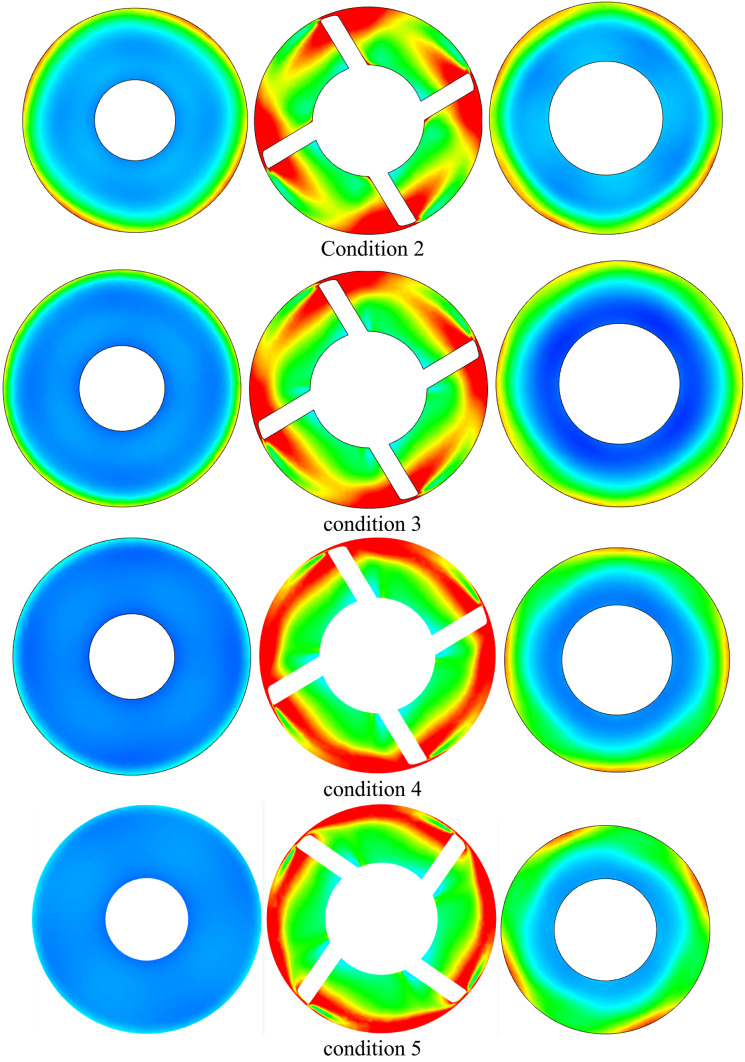
Diffuser contours of axial velocity at various locations in the streamwise direction at conditions conditions.

**Fig. 15:**
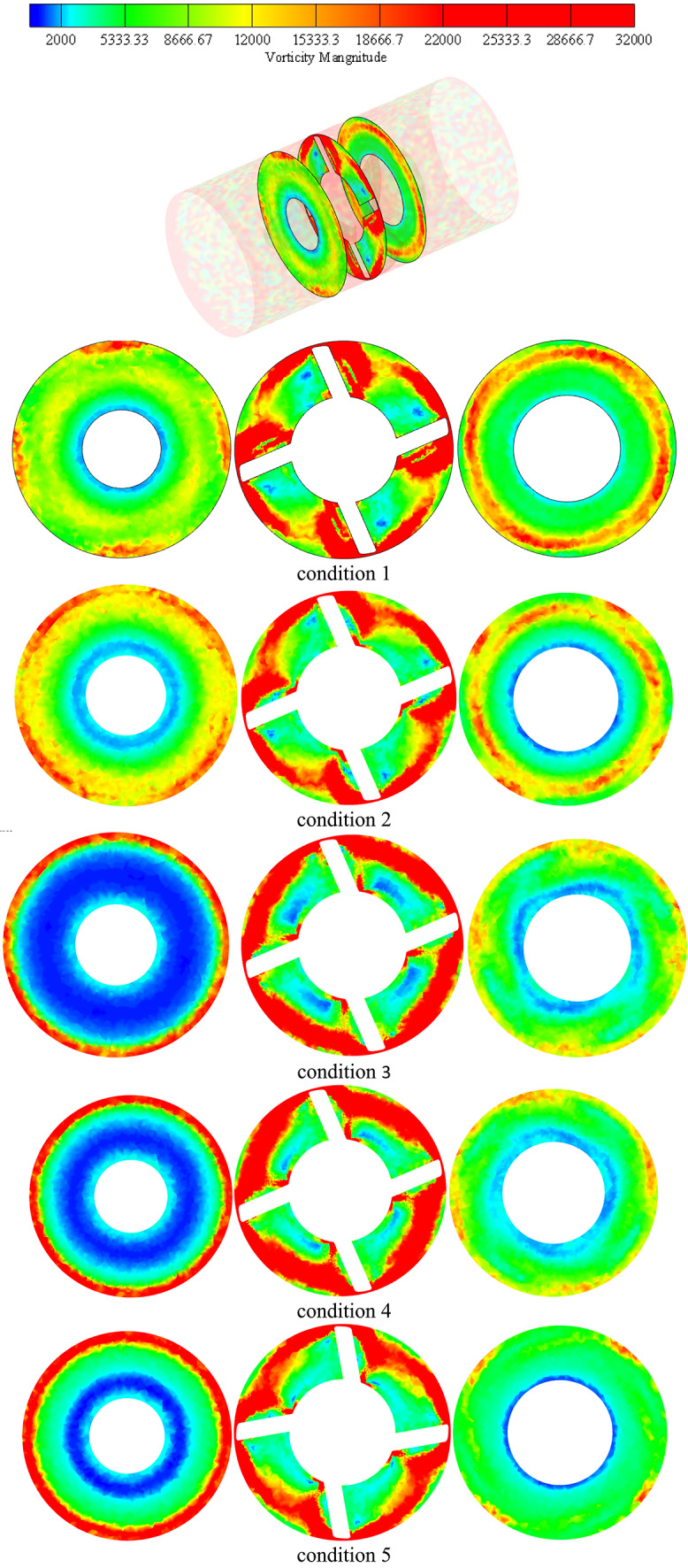
Diffuser axial vorticity contours at various locations in the streamwise direction under conditions flow conditions.

It moves downhill once more to the impeller hub’s vicinal region under upper part-load circumstances, maintaining the string-like shape that extends from the blade leading edge to the impeller outlet area as depicted in Figs. 14 and 15. The next four images illustrate velocity contours and accompanying velocity-colored streamlines within the diffusor flow region under various operating settings in order to analyze the flow dynamics within inter- vane flow channels. Under condition 1 operating circumstances, which is the one nearest to the vaneless area between the diffuser and impeller, shows a small high speed flow at the diffuser’s shroud region and a large region of extremely low flow velocity that extends from the hub to beyond the mid-span region.

**Fig. 16:**
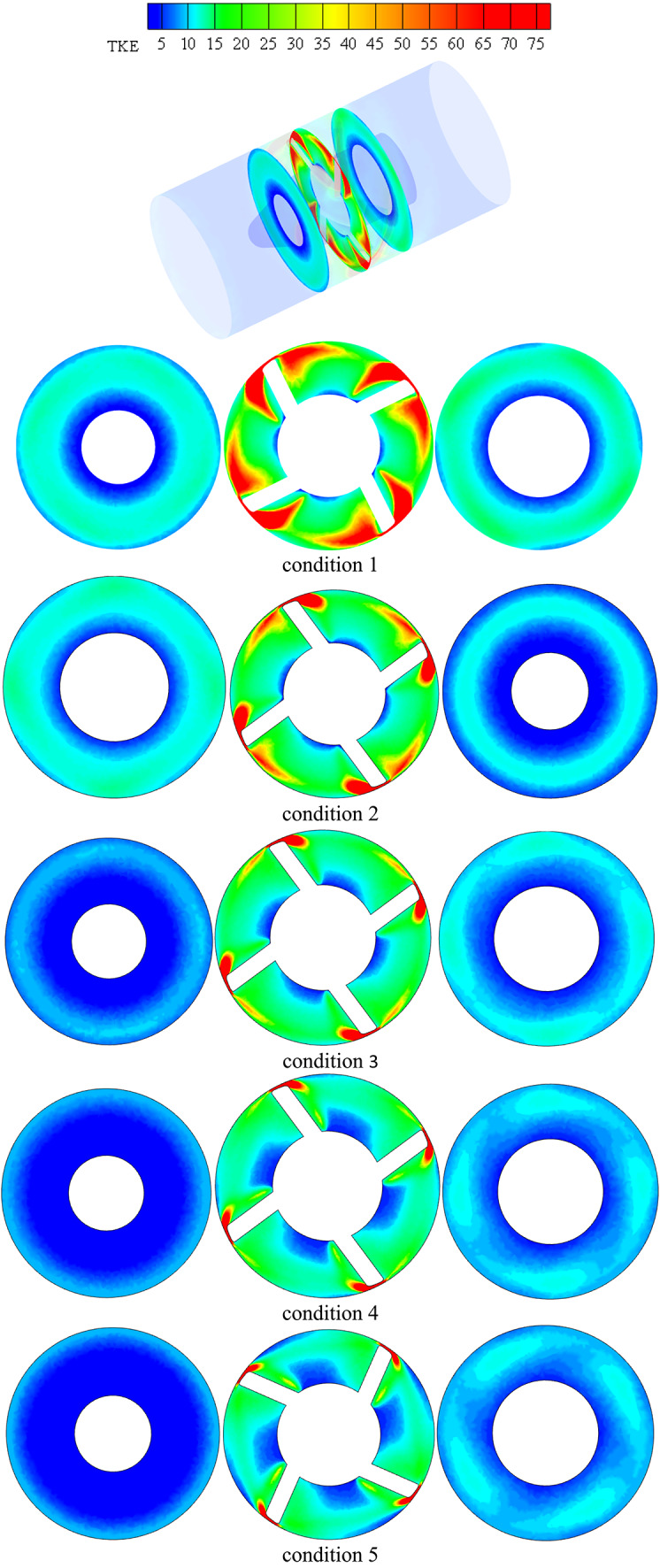
Diffuser axial TKE contours at various locations in the streamwise direction under conditions flow conditions.

In actuality, a wider high speed flow region is attached on the impeller suction surface at this region, which is nearly circumferentially distributed as depicted in Fig. 16. This region extends in the opposite direction of the impeller rotation and eventually reaches the precedent impeller pressure surface on its shroud tip region. Nearly 70% of the flow area is made up of low speed flow in this region. The high-speed flow region on the impeller suction surface separates from the preceding impeller suction surface and expands spanwise downwards at the diffuser mid-distance in the streamwise direction under the identical operating circumstances, pushing the low speed flow region towards the pressure side of the precedent impeller suction surface. The flow area is made up of low speed flow in this region. Tiny high-speed regions remain linked to the pressure and suction surfaces of the vanes while the low speed region shifts to the channel’s center at the diffuser outlet. Over the available flow area is occupied by the low speed flow region at this location.

Large flow vortices and ensuing backflows indicate low flow speed regions, as indicated by corresponding velocity-colored flow streamlines. High speed flows at the shroud and vicinal flow regions are caused by a massive vortex and backflow that distribute in all channels circumferentially and blocks all hub flow regions at the diffuser intake. This pushes the water to small left out flow regions at the shroud. Flow vortices at the diffuser mid-region take up a larger area from the central flow region to the pressure side of one of the bounding blades, leaving the water flowing area at the suction side of another blade and a smaller area in the shroud vicinal region. A double vortex’s immersion at the outlet causes the core region of the channel to be occupied, leaving behind small flowing regions that are connected to the surfaces of both bounding vanes. The flow obstruction caused by the backflows and flow vortices that have formed around the diffuser hub and vicinal flow regions at the diffuser inlet has lessened as machine flow discharge has increased, as can be seen in the remaining Figs. 15, 16. Accordingly, under rated circumstances, there is almost no flow obstruction at the diffuser inlet due to the progressive weakening of flow vorticity at various flow regions inside the diffuser.

### Characteristics of the pressure field

**Fig. 17:**
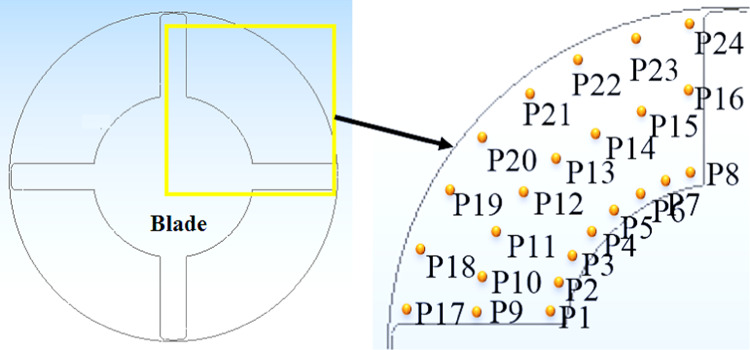
Locations for pressure monitoring in the impeller flow regions.

Several pressure monitors were placed at different points along the pump flow full flow route in order to examine the eventual pressure pulsation features as related to the flow dynamics previously stated. The positioning of the aforementioned pressure monitoring locations within the impeller flow region is shown visually in Fig. 17. The inter-blade flow regions, the suction and pressure sides of the impeller blade between the impeller blades and the distributor’s vanes are the ones that were carefully examined. More specifically, as seen in this Figure, nine monitors in the inter-blade region were placed at three consecutive points streamwise on the impeller’s mid-span plane. Furthermore, while placing pressure monitors on both surfaces of the blade, different spanwise surfaces were taken into account. As seen in this Figure. Each surface had different monitors, for a total of 24 monitors on the blade suction side and blade pressure side.

Figure 17 displays the frequency domain pressure pulsation spectra in the vaneless space for the five operating conditions under investigation. The monitors were shown, though, for clarity’s sake and because pressure pulsation characteristics inside a certain flow region under particular operating circumstances are more likely to show little to no changes. It is discovered that the vaneless space pressure pulsation characteristics change significantly as machine operating conditions change. For example, the dominant frequencies’ pressure pulsation amplitude increased steadily as machine flow conditions dropped from rated conditions to part-load, then dropped to nearly half under deep part-load conditions.

**Fig. 18:**
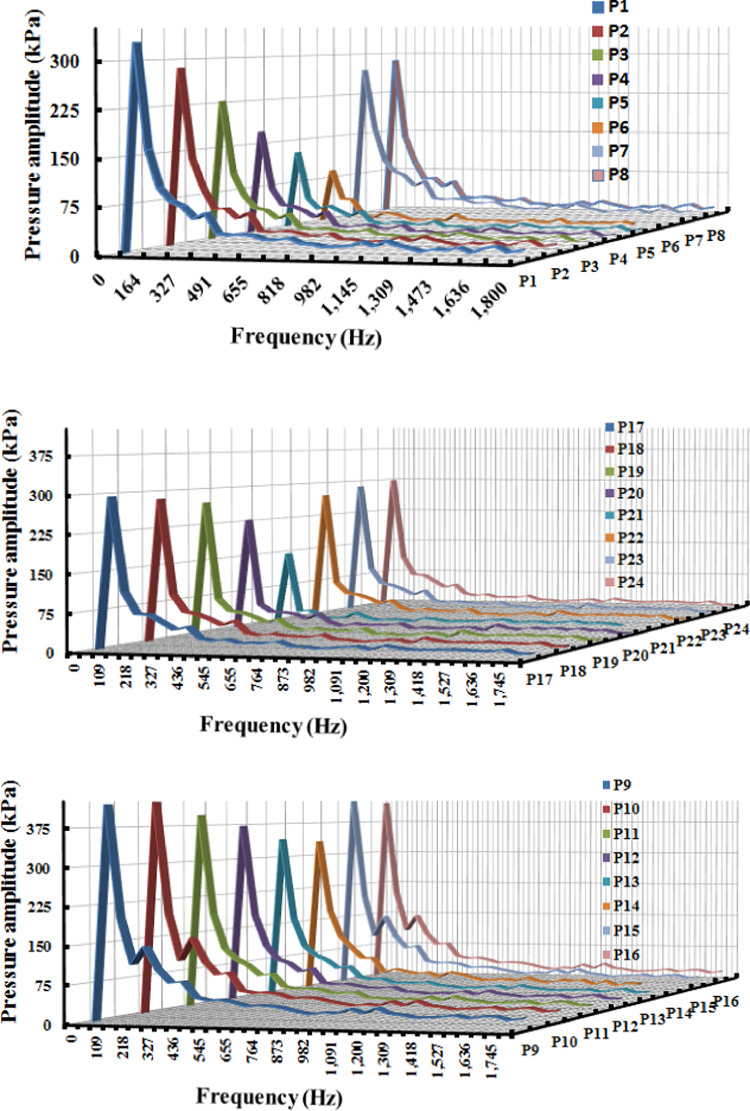
Pressure pulsation characteristics in the frequency domain under condition 3 conditions.

This feature is consistent with the evolution of flow field characteristics outlined above, which showed that as machine inflow dropped, flow structures became more chaotic. The impeller rotational frequency (fn), the dominating frequency fi = 7fn, and its double (2fi) make up the majority of the related pressure pulsation spectrum under rated circumstances. Because the impeller has four blades, the blade passing frequency (BPF) should be 50 Hz, but the impeller rotational speed is 3000 rpm as depicted in Fig. 18. Furthermore, the blade passing frequency shows how the stator blades affect the rotor flow field, whereas the BPF indicates how the continually passing impeller blades disturb the distributor flow field. The reciprocal interactions between the distributor and impeller, commonly referred to as "Rotor–Stator Interactions" or RSI, are represented by BPF. This phenomenon is well known for being a powerful cause of high pressure pulsations in hydraulic equipment, which in extreme circumstances can lead to the development of severe structural vibrations and the eventual breaking or creation of fractures in machine components. The aforementioned dominating frequency component as the examined pump distributor is made up of four vanes. The amplitude of the dominating frequency component, fi, drops to almost when the machine flow conditions change from rated to condition 2 settings.

As the machine operating circumstances deviate from their normal operating conditions, more secondary flows arise, which is linked to the creation. One of the aforementioned linked to the creation (0.2fi) emerged as the dominating frequency under condition 2 circumstances, with other components in the 0.5 fi to 2 fi range coming to the surface. The newly developed blade passing frequency component had the largest pulsation amplitudes under the condition 1 condition. Under deep part-load circumstances, however, the once-dominant component gained back the lead, and the amplitudes decreased to nearly half.

The different operating circumstances stand out for this feature. In general, pressure pulsation spectra were loaded with more low amplitude frequency components (linked to the creation) when machine flow conditions deviate from optimal. As was previously noted, the eventual deterioration of flow unsteadiness inside the machine flow regions is what causes the formation of these frequencies and the ongoing growth in global pressure pulsation amplitudes. Both the RSI-born components and the local flow unsteadiness-born components make up the related pressure pulsation spectra, especially for the last two operating circumstances.

A pressure pulsation was used to try to get a better idea of how machine operating conditions affect the pressure pulsation levels of particular flow regions. In this regard, the distribution mode of pressure pulsation amplitudes at various points within the impeller during the different operating circumstances under investigation is shown in this Figure. The impeller blade pressure and suction surfaces, the vaneless area between the impeller and the distributor, and the impeller inter-blade channel are all considered selected regions. It is typically seen that pressure pulsation amplitudes within the inter-blade flow channels grow streamwise from the impeller intake to the outlet, regardless of the operating parameters taken into consideration. Furthermore, as illustrated once more in this Figure, it is discovered that pressure pulsation amplitudes within the inter-blade flow channels globally increase as machine flow discharge decreases. As a result, deep load conditions displayed the highest pulsation amplitudes, while rated conditions presented the least amount of pressure pulsation. However, compared to other impeller flow regions, the vaneless space region often showed the highest level of pulsation amplitudes. Part-load operating circumstances recorded the largest amplitudes, while the rated conditions still recorded the lowest.

In other words, as seen in this Figure, vaneless space pressure pulsation amplitudes increased as machine flow discharge decreased. They peaked under load circumstances (condition 1) and then decreased once again as part-load conditions became deeper. Both rated and higher part-load circumstances (condition 2 and condition 3) exhibit a more symmetric distribution of pressure pulsation amplitudes, but the latter operating conditions clearly exhibit asymmetric behavior. The appearance of several pressure pulsation frequency spectra, which were thought to be connected to the deterioration of flow unsteadiness under the previous three circumstances, strangely fits in nicely with this.

### Impact of the angle of the impeller blade

**Fig. 19:**
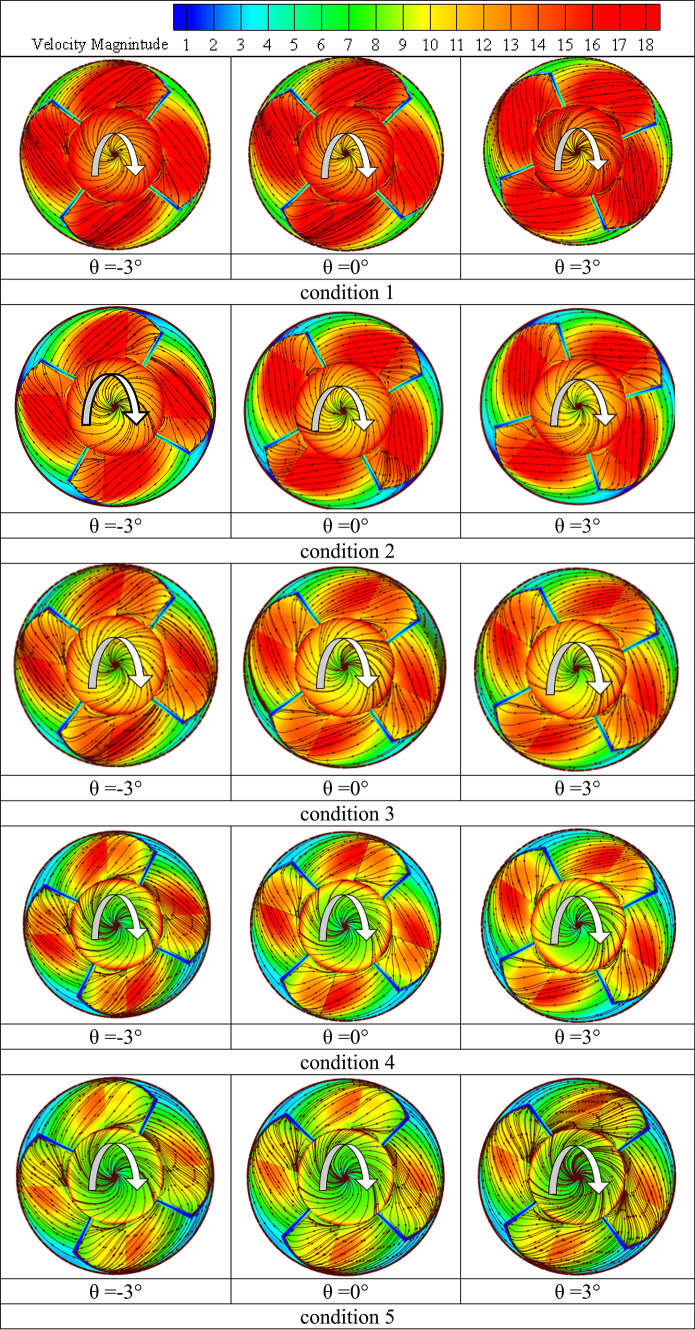
The pressure surfaces of the impeller blade exhibit velocity-colored streamlines under varying conditions flow conditions.

After examining the formation mechanism of pump flow structures and the related pressure pulsation characteristics and distribution modes for various operating conditions, the current study has attempted to examine the impact of impeller blade geometric design on the same. To this end, the impeller blade angle γ was chosen as the testing parameter, and its three values θ = -3°, θ = 0°, and θ =  + 3° were chosen for additional analysis. For different flow conditions load and rated conditions were examined in the investigations of the changes in pump flow dynamics with the varying blade angle as depicted in Fig. 19.

Different flow circumstances the deep-part-load (condition 1) and the rated conditions (condition 3) were examined in investigations on the changes in pump flow dynamics brought about by changing blade angles. Under deep part-load circumstances, Fig. 19 displays velocity-colored streamlines on the suction and pressure surfaces of the blade for three different blade angles: θ =  − 3°, θ = 0°, and θ = 3°. It is clear that changing the impeller blade angle has changed how the flow structures form on both surfaces. For example, adopting a -3° blade angle results in somewhat weaker blade suction side flow vortices at the impeller shroud and vicinal regions. Previously, these vortices were categorized. But when the blade angle increased from -3° to 0° to 3°, these vortices gradually grew stronger and occupied more area. Furthermore, as the impeller blade angle has increased, the high-speed flow region at the leading edge of the blade has expanded. In Fig. 11, associated flow separation shown as wakes. However, as the blade angle increased, the impeller hub-attached vortex flow in the blade pressure side flow field grew stronger.

**Fig. 20:**
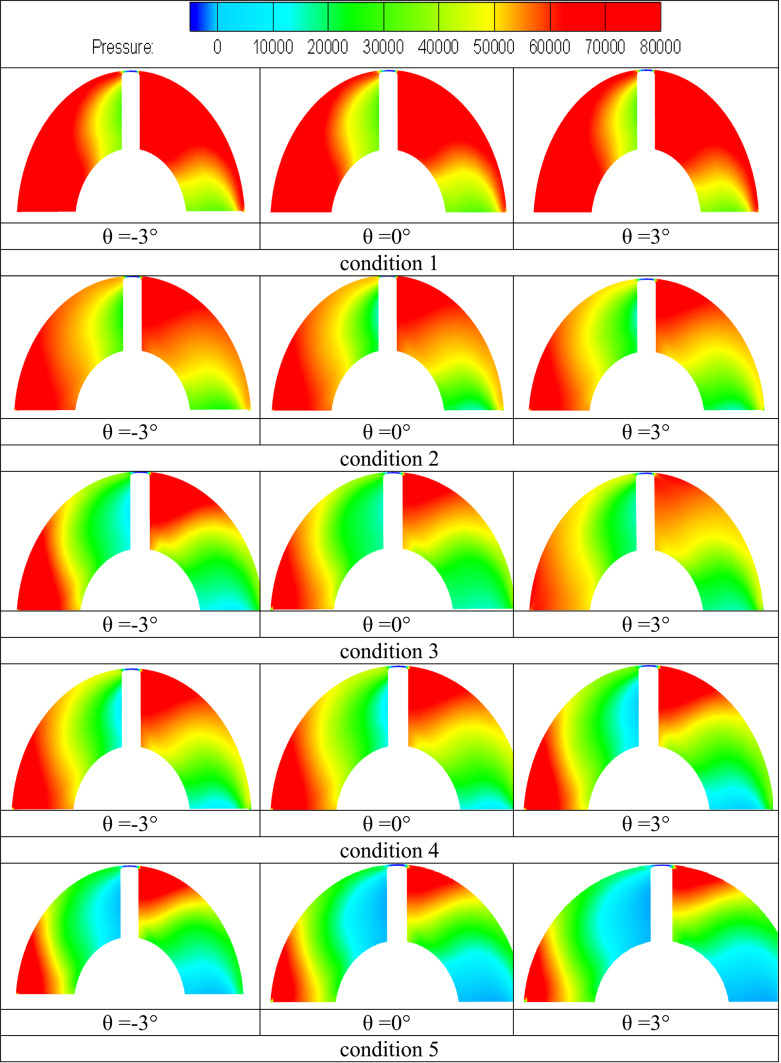
Pressure distribution contours under deep part-load situations on impeller blade suction side.

**Fig. 21:**
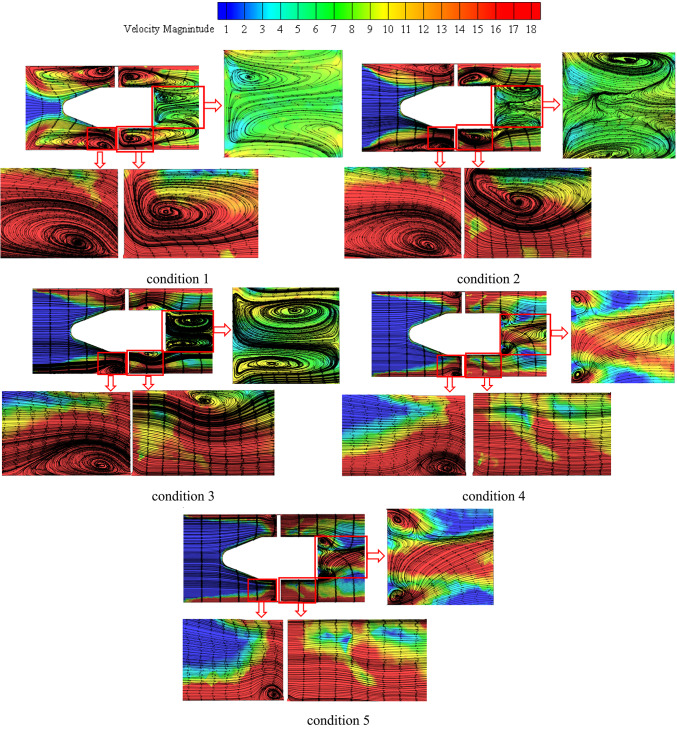
Varying conditions circumstances, axial velocity contours for the three blade angles at the impeller outlet.

According to Fig. 20, blade pressure side ’s hub pressure pulsation amplitudes were the largest when compared to other places along the spanwise direction because of these flow vortices within the hub vicinities. The flow reversal movement back to inter-blade channels was induced by these vortices, which are partially linked to flow unsteadiness within the blade trailing edge vicinal regions. As the impeller blade angle increased, the associated hub backflow region widened. Figures 20 and 21 were utilized to provide further information on the impeller flow and pressure field changes occurring as the impeller blade angle increases in order to bolster the realities presented previously. The pressure distribution mode on both blade suction side and blade pressure side, as well as the subsequent modifications as the blade angle rose, are shown in this Figure. According to the information above, the blade leading-edge low-pressure region on the blade suction side that corresponds to the type A flow separation region has widened in this figure as the blade angle has increased. Additionally, two high pressure regions that correspond to vortex flow and hub backflow regions are seen on the blade pressure side, along with the shroud and hub regions. As the blade angle rose, it was observed that both high pressure regions grew in size and strength. Accordingly,

Figure 21 displays the flow axial velocity contours inside the impeller outlet’s vicinal flow region. different high velocity regions are frequently observed at each of the three blade angles under investigation in this region. Though it doesn’t reach the shroud side, the first is connected to the blade suction side where it stretches from the hub to the over mid-span region. The second, which is fastened to the shroud, stretches from the vicinal region of the first blade’s pressure side to the suction side of the subsequent blade, however it doesn’t quite reach. It was observed that both regions get stronger and thicker as the blade angle increases worldwide. Additionally, for each of the three blade angles under investigation, there is often one low axial velocity region (also known as backflow or negative axial velocity).

This region is connected to the hub and initially separates from the blade pressure side, but it eventually touches it in the vicinal region of the mid-span after extending obliquely towards it. Finally, it is discovered that this region expands, taking up more space at the hub and on the blade pressure side. Under θ = 0° circumstances, it approaches the mid-span region, and for θ =  + 3°, it surpasses it. This is in good agreement with the information provided by previous studied, which states that the hub-attached backflow region expands as the blade angle increases. This also explains how increasing the blade angle strengthens the shroud-attached high axial flow region. The accompanying flow obstruction forces additional water flow towards the shroud region as the hub-attached backflow region thickens. The existent channel flow blockage causes the shroud-attached positive flow to accelerate as the hub backflow region increases. In the same way, the observed negative flow region in the blade suction side ’s vicinal region toward the shroud side under γ = 0° conditions has also resulted in flow obstruction at the same region, which has caused a shroud-attached accelerated water flow region to emerge that touches the blade suction side on its shroud tip region. However, it is discovered that when the impeller blade angle increases, this region vanishes.

**Fig. 22:**
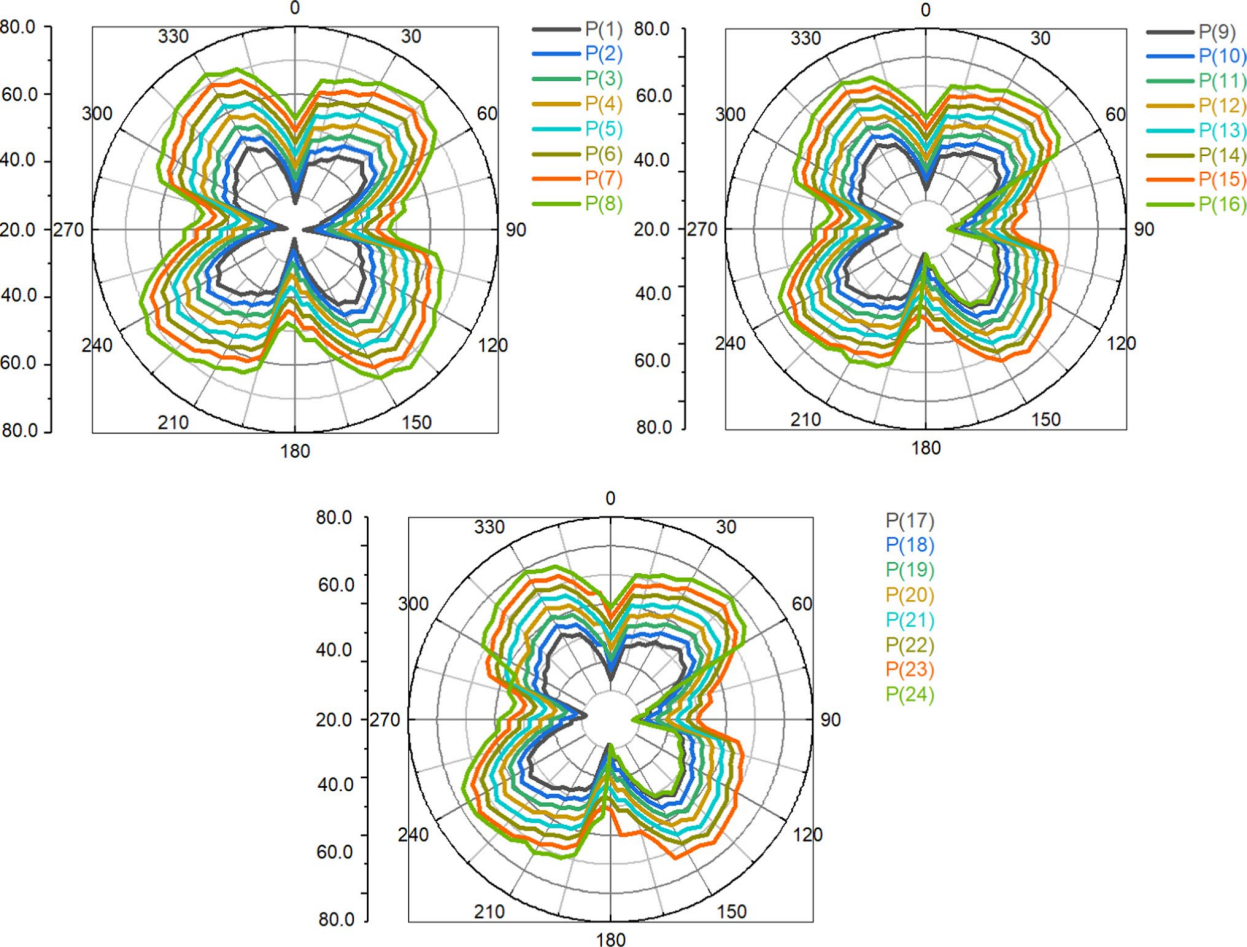
Four impeller blade angles in pressure pulsation distribution at condition 3 operating.

**Fig. 23:**
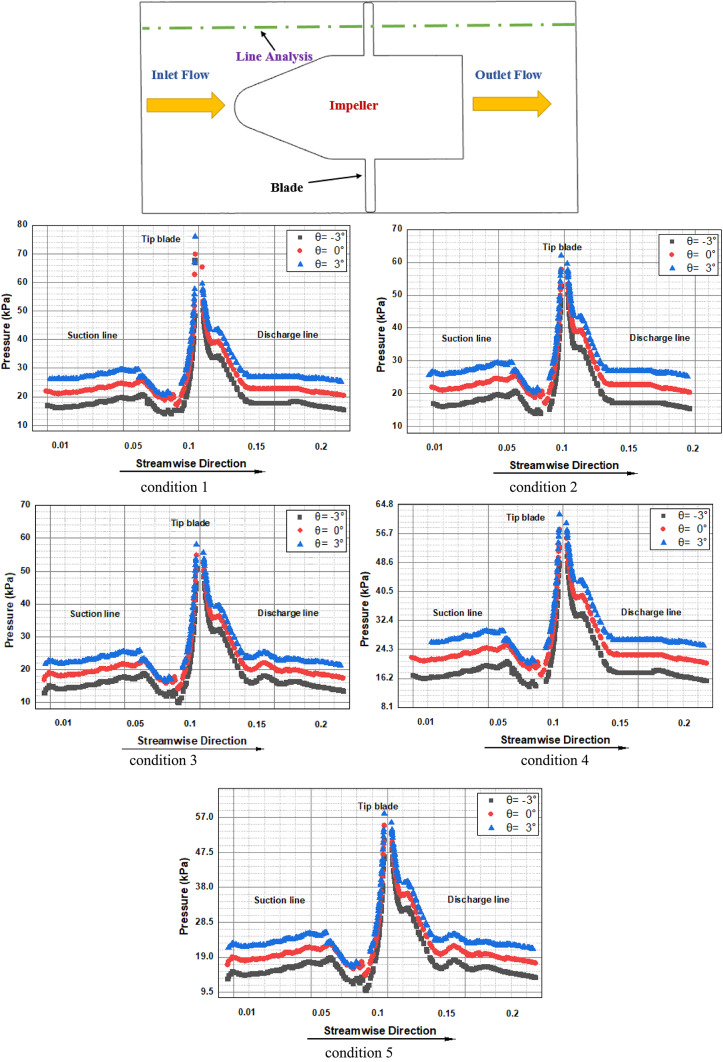
Distribution of pressure pulsations with varying γ values in a streamwise direction Operating conditions.

An attempt was made to investigate corresponding alterations of pressure pulsation characteristics and distribution mode at various components of the examined computational domain after describing pump flow dynamics and eventual changes as the blade angle changed. In this regard, Fig. 22 shows the distribution mode of pressure pulsation amplitudes within the vaneless space flow region for the four blade angles under investigation, taking into account two operating situations: the deep part-load and the rated conditions. Globally, it is discovered that the pressure pulsation level of deep part-load circumstances is about four times more than the rated conditions. Under rated conditions, the pulsation amplitude increases with the blade angle when 24 monitoring locations are positioned equally along the circumference on the mid-span plane. However, under deep part-load conditions, the situation becomes complex, with pulsation amplitudes at different locations varying almost randomly and without any common law. However, the greatest pulsation amplitudes under deep part-load conditions were seen at θ = 0° blade angle. Taking into account the four blade angles, this Figure shows the streamwise pressure pulsation amplitudes distribution mode in the inter-blade. The point at hub were from P1 to P8 are the eight equal monitoring sites that extend from the middle blade impeller region start from P9 to P16. Eight monitoring sites from P17 to P24. were chosen outer the blade region. They are positioned streamwise from the blade impeller ’s inlet to the outlet’s vicinal region on the blade impeller ’s mid-span plane. These consecutive positions were chosen in a streamwise path from the inlet to the outlet’s vicinal, taking into account the distributor’s mid-span plane once again. As a result, pressure monitoring sites were selected throughout the flow passage path blade region. According to this Figure, pulsation amplitudes in the hub region are low and change, regardless of the blade angle or operating condition taken into consideration. They then gradually increase through the impeller channels and vaneless space, reaching the maximum level of pulsation amplitudes within the vanes channels before descending back to the middle region. The pump model with θ =  + 3° has the maximum pressure pulsation amplitudes under rated conditions for most of the flow regions over the range under investigation. θ = 0° and θ =  − 3° exhibit the lowest pressure pulsation levels, respectively. Conversely, under deep part-load circumstances, θ =  − 3° dominated the entire flow passage from the hub through the impeller region to outer blade region, with θ = 0° coming in second. θ =  + 3° once again grabbed the lead in the distributor’s Instagram channels, with θ = 0° coming in second for most regions. Furthermore, several of the details shown in Fig. 23 are clarified in Fig. 23. The middle blade region’s averaged values of recorded pulsation amplitudes under deep part-load circumstances indicate that the largest amplitudes under these conditions were recorded. Thus, in contrast to the condition with graded circumstances, this is the opposite situation. On a worldwide scale, however, the distributor outer blade flow region recorded the highest pressure pulsation amplitudes regardless of the machine operating conditions. Of the four blade angles that were examined.

## Conclusions

This work combined experimental measurements with numerical simulations to investigate the hydraulic behavior and internal flow dynamics of an axial pump operating in pump mode. Particular attention was given to the effects of operating regime and impeller blade stagger angle. Three blade angles (−3°, 0°, and +3°) were examined across a broad range of conditions, extending from low-load operation to deep part-load. The principal outcomes of the study are summarized below.

1. The computational approach exhibited strong agreement with experimental data, with overall discrepancies limited to less than 5%, demonstrating the robustness of the adopted numerical methodology. As the operating point moved away from the design condition toward deep part-load, flow instability increased substantially. The most intense vortical structures and flow distortion developed near the impeller leading edge, along the suction-side shroud, on the pressure-side hub, and at the impeller exit. Conversely, the distributor hub region and guide passage surfaces experienced the greatest pressure and velocity fluctuations, reflecting pronounced rotor–stator interaction in these areas.
2. Detailed analysis of the velocity field showed that low-speed flow occupied nearly 70% of the passage area under part-load conditions. Simultaneously, high-velocity streams formed along the impeller suction surface, separated from upstream blade passages, and spread spanwise toward the mid-region of the diffuser. This redistribution of momentum forced low-velocity fluid toward the pressure side of the preceding blade, degrading flow uniformity and increasing hydraulic losses.
3. The characteristics of pressure pulsations were strongly dependent on the operating condition. As the flow rate decreased from the design point to part-load, pulsation amplitudes increased markedly, reaching a maximum at part-load before diminishing under deep part-load operation. In all examined cases, the pressure spectra were dominated by rotor–stator interaction effects, with the blade passing frequency (BPF) and its harmonics carrying most of the fluctuation energy. This confirms that RSI is the primary driver of unsteady pressure behavior in the pump.
4. Changes in impeller blade angle produced significant modifications to both the internal flow field and pressure pulsation patterns. Increasing the blade angle from − 3° to + 3° consistently intensified flow unsteadiness within the pump. Near the design condition, pressure fluctuations in the vaneless space increased with blade angle, whereas under deep part-load operation the opposite trend was observed. Nevertheless, across all operating conditions, the distributor region exhibited the largest pulsation amplitudes, which rose steadily with increasing blade angle, indicating a high sensitivity of this region to blade geometry variation.
5. Operation away from the design point promoted the formation of secondary flow structures and additional low-frequency excitation components. A dominant low-frequency component near 0.2fi was identified under intermediate conditions, accompanied by supplementary frequencies within the 0.5fi–2fi range. These components are associated with large-scale vortices and unsteady recirculation, signaling a shift from blade-synchronous excitation toward broader, flow-induced instabilities.
6. Despite the breadth of the present analysis, certain limitations remain. The study was confined to pump-mode operation and considered only three discrete blade angles. Effects related to cavitation, fluid–structure interaction, and long-term operational variability were not addressed and may further influence pressure pulsation and vibration responses in practical applications. Additionally, the numerical model assumed rigid walls, neglecting potential structural flexibility that could modify unsteady response amplitudes.

From an engineering standpoint, the findings offer valuable guidance for selecting impeller blade angles and managing off-design operation, particularly with respect to reducing pressure pulsations and controlling flow instability in distributor components. The results indicate that moderate blade angles can achieve a favorable compromise between hydraulic efficiency and unsteady loading, especially under part-load conditions commonly encountered in real systems. Future work will extend the present methodology to turbine-mode operation, enabling direct comparison of flow formation mechanisms between pump and turbine modes. Ultimately, multi-objective optimization techniques will be employed to enhance flow uniformity, suppress unsteady structures, and improve overall efficiency in both operating modes, contributing to the development of high-performance bidirectional hydraulic machines.

## Data Availability Statement

The data supporting this study’s findings are available at the corresponding author’s request.
